# Calibration- and Workload-Aware Machine Learning for PTSD-Focused Triage in Children Referred Following Sexual Abuse

**DOI:** 10.3390/diagnostics16213477

**Published:** 2026-10-24

**Authors:** Sevda Gül, Gökçen Çetinel, Enes Emre Öztürk, Hatice Oksal, Enes Sarıgedik

**Affiliations:** 1 Department of Electronics and Automation, Adapazarı Vocational School, Sakarya University, 54050 Serdivan, Türkiye; 2 Electrical and Electronics Engineering Department, Engineering Faculty, Sakarya University, 54050 Serdivan, Türkiye; gcetinel@sakarya.edu.tr; 3 Sakarya University Technology Development Zones Management Inc. (Sakarya Teknokent), Sakarya University Esentepe Campus, 54050 Serdivan, Türkiye; 4 Department of Forensic Medicine, Faculty of Medicine, Sakarya University, 54050 Serdivan, Türkiye; eneseozturk@gmail.com; 5 Child Advocacy Center, Sakarya Training and Research Hospital, 54050 Serdivan, Türkiye; haticeoksal@gmail.com; 6 Department of Child and Adolescent Psychiatry, Faculty of Medicine, Sakarya University, 54050 Serdivan, Türkiye; esarigedik@sakarya.edu.tr

**Keywords:** post-traumatic stress disorder, childhood sexual abuse, machine learning, probability calibration, clinical triage, clinical decision support

## Abstract

**Background/Objectives:** Early identification of post-traumatic stress disorder (PTSD) after childhood sexual abuse may support timely assessment, but triage requires evaluation of calibration, missed cases, and referral workload. This study assessed whether routinely recorded information could support prioritization for PTSD-focused assessment. **Methods:** This retrospective single-center study evaluated classification of post-event diagnostic outcomes documented by a specialist child and adolescent psychiatrist in 311 forensic referral records. DSM-5 diagnoses were grouped as PTSD versus no recorded post-event psychiatric diagnosis (*n* = 218; PTSD = 106) and PTSD versus other non-PTSD clinical outcomes (*n* = 199; PTSD = 106). Five models were compared. Within each evaluation design, preprocessing, model selection, sigmoid calibration, and threshold derivation were confined to the development partition; each pipeline was applied unchanged to held-out or chronologically later records. **Results:** Histogram-based gradient boosting yielded a held-out ROC-AUC of 0.6190 (95% CI, 0.4472–0.7853) and PR-AUC of 0.6578 in the primary formulation. Its threshold flagged 36 of 44 records and captured 18 of 21 PTSD cases (sensitivity, 0.8571; specificity, 0.2174). LightGBM yielded a ROC-AUC of 0.6441 (95% CI, 0.4475–0.8125) and PR-AUC of 0.6805 in the secondary formulation. Its threshold flagged 30 of 40 records and captured 18 of 21 PTSD cases (sensitivity, 0.8571; specificity, 0.3684). Calibration reduced Brier scores to 0.2537 and 0.2401, but the calibrated primary model remained slightly worse than the prevalence-only reference (Brier skill score, −0.0165; calibration slope, 0.4276). Temporal ROC-AUC values were 0.6358 and 0.6767, with high workloads. **Conclusions:** Routinely recorded information provided modest discrimination, and high case capture required assessment of most records. External validation, prospective workflow evaluation, and local recalibration are required before clinical use.

## 1. Introduction

Childhood sexual abuse is a serious public health problem associated with developmental, psychiatric, familial, and social consequences that may persist across the life course [1,2]. Recent global estimates indicate that sexual violence during childhood affects hundreds of millions of individuals, with a particularly high burden among girls and women, although boys and men are also substantially affected [2]. Children referred following sexual abuse often present within a complex clinical context in which trauma exposure, developmental vulnerability, family circumstances, and legal or forensic processes intersect. Post-traumatic stress disorder (PTSD) is an important potential outcome because persistent trauma-related symptoms can impair emotional regulation, school functioning, interpersonal relationships, and longer-term psychosocial adjustment. Its occurrence is nevertheless heterogeneous. Meta-analytic evidence indicates that PTSD rates vary by trauma type and sex, with higher rates particularly among girls and children exposed to interpersonal trauma [3]. Other children may present with different psychiatric outcomes or no diagnosable post-event disorder at the time of assessment.

This heterogeneity creates an operational as well as a diagnostic challenge. Every child referred following sexual abuse should have access to comprehensive, trauma-informed psychiatric evaluation and appropriate follow-up. When immediate specialist capacity is constrained, however, services may also need to determine which children require timely or additional PTSD-focused assessment. Any model developed for this purpose must remain subordinate to specialist judgment. Its role is not to exclude children from care or assign an automated diagnosis, but to provide a transparent prioritization signal that can be interpreted within the broader clinical and forensic context.

Machine learning (ML) has increasingly been explored in psychiatry because it can represent nonlinear and multidimensional relationships among demographic, developmental, psychosocial, and clinical variables [4]. Previous studies have used early post-trauma information and routinely collected data to predict PTSD screening status, clinical course, and symptom trajectories [5,6,7,8]. A recent systematic review and meta-analysis of 23 ML studies found that 48% were at high risk of bias and the remainder had unclear risk; substantial heterogeneity and limited validation further constrained the applicability of reported performance [9]. These findings reinforce a broader concern in clinical prediction research: discrimination alone does not establish clinical usefulness [10,11]. A model with an apparently acceptable receiver operating characteristic area under the curve (ROC-AUC) may still yield poorly calibrated probabilities, miss clinically important cases, or generate an assessment workload that cannot be implemented in practice. Calibration, threshold selection, and validation under plausible changes in case mix or time are therefore central to responsible clinical evaluation.

Evidence specific to children referred following sexual abuse remains limited. Ucuz et al. developed an artificial-intelligence-based decision-support system for depression and PTSD, while Gokten and Uyulan used a random forest classifier to predict these outcomes in sexually abused children and adolescents [12,13]. These studies demonstrated the feasibility of ML-based prediction in this population, but their primary emphasis was classification performance. The implications of calibrated risk estimates, threshold-specific referral workload, false-positive assessments, missed PTSD cases, and performance in chronologically later records remain less clear. This leaves an important gap between statistical prediction and the evaluation of a triage approach that could be considered within clinical workflow.

Accordingly, the present study evaluated whether routinely recorded information could support PTSD-focused prioritization among children referred following sexual abuse. Two clinically distinct formulations were examined: PTSD versus no recorded post-event psychiatric diagnosis and PTSD versus other non-PTSD clinical outcomes. Model evaluation considered ROC-AUC, average precision (hereafter referred to as PR-AUC), probability calibration, development-derived operating thresholds, referral workload, captured and missed PTSD cases, and internal temporal performance. By linking model performance to the operational consequences of threshold use, the analysis was intended to clarify both the potential and the limits of the available information in a realistic triage setting.

## 2. Materials and Methods

**Figure 1:**
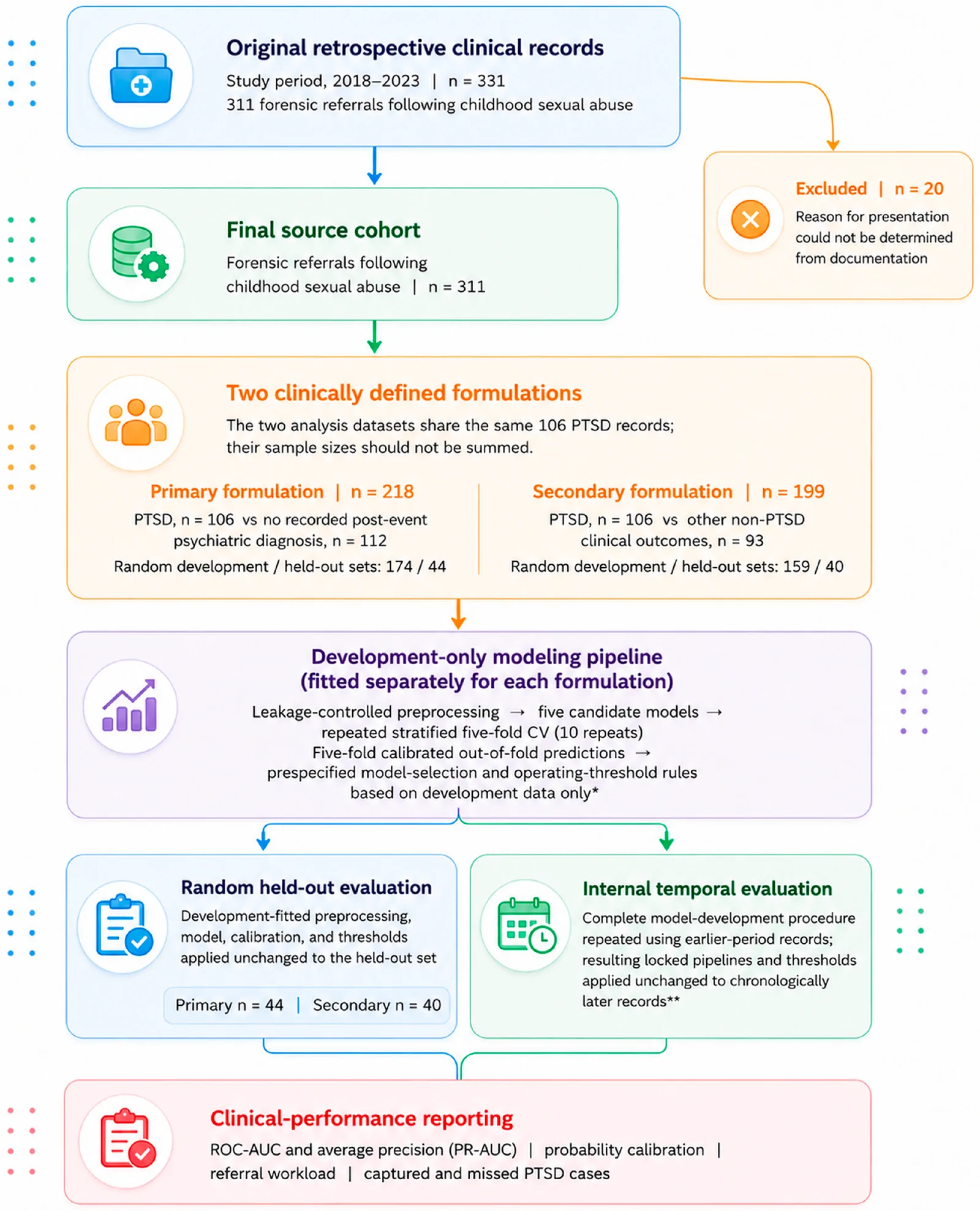
Study workflow and analytical design. The single asterisk (*) indicates that data-adaptive preprocessing, model selection, calibration, and threshold derivation were restricted to development data. The double asterisk (**) indicates that the full development procedure was repeated using earlier-period records before application to later records. Temporal evaluation was internal and retrospective.

The study workflow is summarized in Figure 1. Cohort construction and diagnostic outcome reconstruction preceded separate leakage-controlled development procedures for the random held-out and internal temporal evaluations. Within each evaluation design, preprocessing, model selection, probability calibration, and threshold determination were restricted to the relevant development data before the resulting pipeline was applied to the corresponding evaluation records.

### 2.1. Study Design and Diagnostic Formulation

This single-center retrospective study used routinely collected records from the Child and Adolescent Psychiatry Clinic of Sakarya University during the 2018–2023 study period. All 331 archived records from this period were screened. Records documenting forensic referral following sexual abuse and containing sufficient information to establish eligibility and reconstruct the diagnostic outcome were included. Twenty archived records were excluded because the reason for presentation could not be determined from the available documentation. Although the aggregate number and general reason for exclusion were identified, an individual-level exclusion log was not retained. More detailed exclusion criteria, therefore, could not be reported, and selection bias during cohort assembly cannot be completely ruled out.

The final source cohort comprised 311 forensic referrals. No record was excluded on the basis of the recorded post-event psychiatric outcome. Pre-existing mental disorders were not an exclusion criterion. Otherwise, eligible forensic referral records were retained irrespective of documented psychiatric history. No a priori sample-size calculation was performed. All eligible records available during the study period were included; the sample size was therefore determined by record availability rather than a prespecified power or precision target. Given the limited numbers of records and PTSD events, the discrimination, calibration, and operating-threshold analyses were interpreted as exploratory. Because a stable person-level identifier was not consistently available in the analytical files, multiple records from the same child could not be reliably identified. Both random and chronological partitioning were performed at the record level; child-level separation between development and evaluation samples could therefore not be verified.

All clinical variables and post-event psychiatric diagnoses were recorded and evaluated by a specialist child and adolescent psychiatrist during routine forensic psychiatric assessment. PTSD and comparator diagnoses were assigned according to the Diagnostic and Statistical Manual of Mental Disorders, Fifth Edition (DSM-5), based on clinical interview and review of the clinical record [14]. The documented specialist assessment served as the retrospective clinical reference standard. No additional structured research interview was administered for research purposes.

The diagnostic outcome represented clinician-recorded DSM-5 PTSD. Diagnoses were not retrospectively reassigned to ICD-11 PTSD or complex PTSD because the available records did not contain the systematically documented symptom information required for reliable criterion-based reassessment. PTSD symptom duration and longitudinal course could not be systematically characterized from the analytical data. The interval between the event and examination was used solely as an event-timing variable and was not interpreted as the duration of PTSD symptoms.

Outcome categories were reconstructed from the two post-event diagnosis fields documented in the clinical records. This rule-based procedure did not generate new clinical diagnoses and was used solely to harmonize psychiatrist-recorded diagnostic labels. The standard English abbreviation PTSD, the Turkish abbreviation TSSB, and the documented variants TSBB and PTSB were harmonized as PTSD labels. Records documenting the absence of a post-event psychiatric diagnosis and containing no diagnostic secondary entry were assigned to the no-diagnosis group. The remaining non-PTSD records were assigned to the other-clinical-outcome group.

Two binary formulations were defined. The primary formulation compared 106 PTSD records with 112 records showing no post-event psychiatric diagnosis, resulting in a total sample of 218. The secondary formulation compared the same 106 PTSD records with 93 records showing other non-PTSD clinical outcomes, resulting in a total sample of 199. The comparator groups were mutually exclusive and, together with the shared PTSD group, covered all 311 source records. The secondary formulation was interpreted as a heterogeneous clinical-comparator analysis rather than as a disorder-specific differential diagnosis model.

For descriptive clinical characterization, both post-event diagnostic fields were reviewed to identify additional recorded diagnoses within the PTSD group and characterize the other-clinical-outcome group. Repeated mentions of the same diagnosis within a record were counted once, whereas different diagnoses recorded in the same record were summarized separately. These summaries described the available clinical documentation without generating new diagnoses or changing the binary outcome assignments.

For each formulation, the binary outcome y_i_ was coded as 1 for PTSD and 0 for the comparator group. The modelling task was retrospective classification of clinician-recorded post-event diagnostic status rather than prospective prediction of PTSD onset. The model output represented the estimated probability of membership in the recorded PTSD group, as defined in Equation (1), where X_i_ denoted the predictor vector for record i. Calibration and threshold analyses explored the potential implications for PTSD-focused assessment workload; they did not constitute prospective validation of a clinical triage workflow.

(1) $${\hat{p}}_{i}=P(y_{i}=1|X_{i})$$

The study was approved by the Sakarya University Faculty of Medicine Non-Interventional Clinical Research Ethics Committee (approval no. E-71522473-050.01.04-300081-303, 2023). Owing to the retrospective study design, the requirement for individual informed consent was waived by the ethics committee.

### 2.2. Predictor Construction and Preprocessing

Predictors were drawn from routine clinical records and were intended to represent information available at or near the initial forensic psychiatric assessment. Because variable-level documentation times could not be verified retrospectively, availability before diagnostic assignment could not be confirmed for every predictor. The 22 candidate predictors covered seven clinically interpretable domains: child characteristics, maternal characteristics, paternal characteristics, family context, event timing, abuse characteristics, and perpetrator relationship. The complete predictor content is summarized in Table 1. Post-event diagnosis fields, the reconstructed outcome label, variables directly derived from diagnostic information, and personal or administrative identifiers were excluded from the model input matrix. Raw examination date was not used as a predictor and was retained solely for temporal partitioning.

The source clinical data contained mixed date, year, age, categorical, and free-text formats. Examination dates and birth information were standardized before clinically interpretable age variables were derived. Parental age fields recorded as birth dates, birth years, or direct ages were converted consistently relative to the examination date. Expressions describing the interval between the event and examination in days, weeks, months, or years were converted to a common yearly scale and were used when deriving age at the event. Ambiguous values were retained as missing rather than being replaced by assumed corrections.

Categorical fields were consolidated using prespecified, clinically meaningful categories before model encoding. Missing continuous and discrete numerical values were imputed using the median estimated exclusively within the corresponding training partition. Explicitly documented unknown categorical values were retained as a separate category, whereas remaining blank categorical values were imputed using the most frequent category estimated within the corresponding training partition. Categorical predictors were subsequently transformed using one-hot encoding, with previously unseen categories ignored when the fitted encoder was applied to validation, held-out, or temporally later records. After harmonization, categorical unknown values were represented explicitly, and no blank categorical entries remained in the predictor matrices used for these analyses.

Continuous predictors were standardized for L2-regularized logistic regression. Tree-based models were fitted using the imputed and encoded feature matrix without additional numerical standardization. Prespecified rule-based data cleaning, clinical range checks, duration conversion, and category harmonization were applied deterministically before data partitioning and did not use outcome information. In contrast, data-adaptive imputation, numerical standardization, and one-hot encoding were fitted exclusively within the corresponding development or cross-validation training partition and were subsequently applied unchanged to validation, held-out, or temporally later records.

Predictor content, data types, and formulation-specific missing or unknown ranges are summarized in Table 1. Percentages were calculated separately within each diagnostic formulation, and each range represents the minimum and maximum missing or unknown proportion among predictors in the corresponding domain.

Missingness was generally limited but reached 31.7% (69/218) and 25.6% (51/199) for paternal age in the primary and secondary formulations, respectively. Deception was missing or recorded as unknown in 22.0% and 23.1% of records, while the corresponding proportions for threat were 18.8% and 19.1%. Sex and biological/adopted status were complete in both formulations. All data-adaptive imputation parameters were estimated exclusively within the corresponding training partitions.

### 2.3. Machine-Learning Models

Five supervised learning models were evaluated to represent linear, bagging-based, and boosting-based approaches suitable for tabular clinical data. L2-regularized logistic regression was included as a transparent reference model. Random forest was evaluated as a bagging-based ensemble capable of representing nonlinear relationships and interactions. Histogram-based gradient boosting, XGBoost, and LightGBM were included as gradient-boosting approaches with different tree-construction and optimization strategies [15,16,17,18].

The purpose of comparing these models was not to treat algorithmic complexity as an objective in itself, but to determine whether nonlinear ensemble methods provided a practically meaningful improvement over a regularized linear baseline. Model selection was therefore based on the prespecified clinical operating objective rather than exclusively on the highest ROC-AUC or PR-AUC.

Each diagnostic formulation was divided into development and held-out test sets using a stratified 80:20 split with a fixed random seed of 42. The primary formulation yielded 174 development records and 44 held-out test records, while the secondary formulation yielded 159 development records and 40 held-out test records. The held-out sets were not used during preprocessing, model selection, calibration fitting, or operating-threshold derivation.

Model stability within the development data was assessed using repeated stratified five-fold cross-validation with 10 repeats, yielding 50 validation estimates for ROC-AUC and PR-AUC. Separately, calibrated out-of-fold probabilities for model and threshold selection were generated exclusively within the development set using five-fold stratified outer cross-validation. Within each outer training fold, sigmoid calibration was fitted using CalibratedClassifierCV with up to five internal cross-validation folds, and calibrated probabilities were generated only for the corresponding outer validation fold. The final calibrator was subsequently fitted using the full development set and applied unchanged to the held-out test set. Held-out outcomes were not used for calibration fitting, model selection, or threshold derivation.

A prespecified screening-oriented selection rule was applied. Candidate models satisfying a development-stage sensitivity requirement of at least 0.85 were compared according to specificity. When more than one model provided the same specificity, ties were resolved sequentially according to higher sensitivity, lower Brier score, and higher PR-AUC. Once a model and operating threshold had been selected, both were locked and applied unchanged to the held-out test set. Held-out outcomes were used only for final performance estimation and did not influence model or threshold selection.

Automatic hyperparameter optimization was not performed. Each algorithm was evaluated using one analyst-specified fixed configuration, summarized in Table 2. These settings were pragmatic implementation choices for a reproducible comparison of specified pipelines; no systematic optimization criterion was used to establish the individual parameter values as optimal. The configurations were fixed before final evaluation and applied consistently across both diagnostic formulations and the random and chronological evaluation designs. Parameters not explicitly overridden retained the defaults of the reported library versions.

All candidates used the same predictor set, development and evaluation partitions, cross-validation splits, sigmoid calibration strategy, threshold grid, and development-stage selection criteria. Numerical standardization was applied to logistic regression, and model-specific class-weighting procedures are described below. This common evaluation protocol supported procedural consistency, but it did not equalize model capacity or establish the best achievable performance of each algorithm after tuning. The results therefore compare the reported configurations rather than optimized algorithm families.

Class weighting was applied according to model type. Class 0 denoted the relevant comparator group and class 1 denoted PTSD. Logistic regression used class weights calculated as *w_c_* = *n*/(2*n_c_*), whereas XGBoost and LightGBM used scale_pos_weight = n_negative/n_positive. Histogram-based gradient boosting was fitted without explicit class weighting. Under balanced_subsample, random-forest class weights were recalculated from the bootstrap sample used to construct each tree and therefore had no single fixed value. All other data-dependent class weights were recalculated within the corresponding cross-validation training partition. Although subsample was set to 0.90 for LightGBM, row subsampling remained inactive because subsample_freq was retained at 0.

At every resampling stage, all data-adaptive preprocessing operations were fitted exclusively within the corresponding training partition. The fitted preprocessing pipeline, model, calibration procedure, and operating threshold were subsequently applied without modification to the relevant validation or held-out partition.

### 2.4. Evaluation Framework

Model evaluation was aligned with the intended clinical triage use and therefore addressed discrimination, calibration, threshold-specific clinical performance, and referral workload. Discrimination was quantified using ROC-AUC and average precision, reported here as PR-AUC. ROC-AUC summarized rank separation across possible thresholds. PR-AUC was calculated using sklearn.metrics.average_precision_score rather than by trapezoidal integration of the precision–recall curve. Because PTSD prevalence was close to one half in both diagnostic formulations, PR-AUC was interpreted as a complementary retrieval measure rather than solely as a metric for marked class imbalance [19].

For the held-out and temporal evaluations, ROC-AUC and PR-AUC point estimates were calculated directly from the observed outcomes and uncalibrated predicted probabilities. Calibrated probabilities were used for threshold derivation and threshold-specific clinical evaluation. Ninety-five percent confidence intervals were estimated using 1000 nonparametric bootstrap resamples. Only resamples containing both outcome classes were retained for interval estimation. The bootstrap distribution was used solely to estimate the confidence intervals; its mean was not substituted for the observed point estimate. Clopper–Pearson exact binomial confidence intervals for sensitivity, specificity, positive predictive value, and negative predictive value were calculated using statsmodels. All confidence intervals were interpreted descriptively because formal pairwise superiority testing was not prespecified.

An exploratory full-pipeline bootstrap assessed development stability. Within each formulation, 100 development samples were drawn with replacement using a fixed pseudorandom seed (random_state = 20261017). Each replicate repeated data-adaptive preprocessing, nested out-of-fold sigmoid calibration for all five candidates, Rule A model and threshold selection, and refitting using the original hyperparameters and selection rules. Bootstrap copies of each source record remained together within validation and calibration folds. Selected workflows were evaluated on the original held-out records and, separately, on independently resampled held-out records. Model-selection frequencies and candidate-specific threshold and performance distributions were summarized using medians and interquartile ranges. These analyses were conditional on the original random partition and did not replace the original point estimates or establish sample adequacy or external validity.

Exploratory, non-prespecified tests of ROC-AUC = 0.50 used two-sided Wald statistics with DeLong’s nonparametric variance estimator and the same uncalibrated evaluation probabilities as the reported ROC-AUC estimates [20]. Holm adjustment was applied across all 20 tests (five models, two formulations, and two evaluation designs) [21]. Statistical significance was assessed at an adjusted *p* < 0.05. These tests did not influence model selection, calibration, or operating thresholds, and no pairwise model-superiority tests were performed.

Post hoc permutation importance was evaluated for the 22 original predictors in the development-selected primary HGB and secondary LightGBM models [15]. Each predictor was independently permuted 100 times within the corresponding held-out dataset using a fixed pseudorandom seed (random_state = 42), before the fitted preprocessing steps. Preprocessing, model parameters, and calibration were held unchanged. Importance was defined as the reduction in raw-score ROC-AUC and the increase in calibrated Brier score relative to the unpermuted data. Means and standard deviations summarized permutation variability rather than sampling uncertainty. The analysis was not used for feature selection, model tuning, or threshold revision.

A post hoc sensitivity analysis compared four missing-data strategies in the original random partitions: numerical median imputation with categorical unknowns retained (A); numerical mean imputation (B); numerical median imputation with categorical unknowns replaced by the training-partition mode (C); and strategy A with numerical missingness indicators (D). The selected model families and fixed hyperparameters were retained, and each model and its sigmoid calibration were refitted using development data only. The same held-out records were used across strategies. Discrimination and calibration metrics were compared descriptively; model selection and threshold derivation were not repeated. The analysis neither established the missingness mechanism nor propagated missing-value uncertainty through multiple imputations.

A post hoc subgroup analysis examined sensitivity and false-negative rates by age at the event (<12, ≥12, or unavailable), recorded sex, and recorded abuse type. The original calibrated predictions and development-derived Rule A thresholds were retained separately for both formulations and evaluation designs; no model was refitted, and no subgroup-specific threshold was derived. For each subgroup, record and PTSD counts, true positives, false negatives, sensitivity, false-negative rate, and pointwise exact 95% confidence intervals were reported. Measures were not estimated when a subgroup contained no PTSD records. Results were descriptive, and no formal subgroup comparisons or claims of equivalent performance or algorithmic fairness were made.

At operating threshold t, record i was flagged for additional PTSD-focused assessment when ${\hat{p}}_{i}$ ≥ t. True positives (TP), false positives (FP), true negatives (TN), and false negatives (FN) were used to calculate sensitivity, specificity, positive predictive value, and negative predictive value. Clinical workload was defined as the number of records requiring downstream PTSD-focused assessment:

Workload(t) = TP(t) + FP(t)(2)

Workload rate and referral reduction relative to an assess-all strategy were calculated as follows, with *N* denoting the number of records in the relevant evaluation set:

Workload rate(t) = Workload(t)/N(3)

Referral reduction(t) = 1 − Workload(t)/N(4)

A fixed threshold of t = 0.05 was retained only as a deliberately permissive reference condition. It was neither treated as the principal clinical operating threshold nor used to select the final model. Its purpose was to demonstrate the workload generated by applying a uniformly low screening threshold to calibrated probabilities.

Candidate thresholds ranging from 0.01 to 0.99 in increments of 0.01 were evaluated using calibrated out-of-fold predictions generated exclusively within the development data. Three operating rules were prespecified. Rule A represented a screening-oriented regime and selected, among thresholds satisfying sensitivity ≥0.85, the threshold with the highest specificity. Rule B1 represented a precision-oriented regime and selected the threshold that maximized positive predictive value. Rule B2 represented a specificity-constrained regime and selected, among thresholds satisfying specificity ≥0.85, the threshold with the highest positive predictive value. For Rule A, ties were resolved by higher sensitivity, higher positive predictive value, and then the higher threshold. For Rules B1 and B2, ties were resolved by higher specificity, higher sensitivity, and then the higher threshold. After derivation, the selected thresholds were locked and applied unchanged to the held-out test set; held-out outcomes were not used to optimize, revise, or replace them.

The sensitivity requirement of 0.85 was an analyst-defined exploratory constraint intended to prioritize identification of recorded PTSD cases while allowing some reduction in assessment workload. It was not established as a clinically acceptable minimum sensitivity, and it should not be interpreted as evidence that missing up to 15% of PTSD cases would be acceptable. This requirement concerned development-stage sensitivity and was distinct from the probability threshold applied to individual records.

In an additional post hoc analysis, the sensitivity requirement was increased to 0.90 and 0.95. For each requirement, candidate-specific thresholds and model selection were recalculated using the original calibrated development out-of-fold predictions and the original selection rules. The resulting model–threshold combinations were evaluated using their previously saved held-out predictions. No additional models were fitted, and held-out results were not used to replace the original sensitivity requirement. These comparisons were descriptive and did not establish clinical acceptability.

Probability calibration was evaluated because predicted probabilities may be interpreted as risk estimates when used for clinical prioritization. Sigmoid calibration was fitted exclusively within the development stage using the nested cross-validation procedure described above [22]. Raw and calibrated probabilities were compared using the Brier score, calibration-in-the-large, reported as the calibration intercept, and calibration slope. Calibration-in-the-large was estimated using a binomial generalized linear model in which the logit-transformed predicted probability was included as an offset and a single intercept was estimated. The calibration slope was estimated using a separate binomial generalized linear model containing an intercept and the logit-transformed predicted probability as a covariate. Predicted probabilities were clipped to the interval [10^−6^, 1–10^−6^] before logit transformation. Threshold selection and threshold-specific clinical evaluation were based on calibrated probabilities.

Probability error was quantified using the Brier score defined in Equation (5).

(5) $$\mathrm{Brier}=(1/N)\;{\Sigma_{i=1}}^{N}\;{\left({\hat{p}}_{i}-y_{i}\right)}^{2}$$

The change in Brier score following calibration was calculated using Equation (6).

ΔBrier = Brier(raw) − Brier(calibrated)(6)

A positive ΔBrier indicated a reduction in average probability error. To contextualize calibrated Brier scores, the development-set PTSD prevalence was applied as a constant probability to held-out records. The Brier skill score (BSS) was calculated relative to this prevalence-only reference using Equation (7).

BSS = 1 − Brier(calibrated)/Brier(reference)(7)

Positive BSS values indicated improvement over the prevalence-only prediction, whereas negative values indicated no improvement over the constant-probability reference. Calibration findings were interpreted as exploratory because of the evaluation sample sizes. These comparisons did not influence model or threshold selection.

Internal temporal evaluation was performed using examination date to assess model behavior under chronological separation. The cutoff date was selected algorithmically rather than according to an externally defined clinical or organizational milestone. Among candidate examination dates that retained at least five observations and both outcome classes in the earlier and later periods, the date producing a later-period proportion closest to the prespecified 20% holdout fraction was selected.

This procedure yielded 19 July 2018 for the primary formulation, with 174 earlier development records and 44 later test records, and 13 July 2018 for the secondary formulation, with 159 earlier development records and 40 later test records. The equality of these sample sizes with the corresponding random 80:20 partitions reflected the common target holdout fraction; record assignment remained chronological and differed from that in the random partitions. All records included in the final formulations had valid examination dates.

Temporal model selection, probability calibration, and operating-threshold derivation were performed using only the earlier-period development data. The resulting preprocessing pipeline, selected model, calibration procedure, and operating threshold were applied unchanged to the later-period records. No performance metric from the later-period data was used during model or threshold selection. This analysis assessed retrospective classification in chronologically later records from the same center and did not constitute external validation or prospective prediction of PTSD onset.

Model development and the original performance analyses were conducted in Python 3.10.18 on Windows 10 using pandas 2.3.3, NumPy 2.2.6, scikit-learn 1.7.2, XGBoost 3.1.2, LightGBM 4.6.0, statsmodels 0.14.6, and Matplotlib 3.10.9. The additional exploratory AUC tests used saved evaluation probabilities in Python 3.12.14. The bootstrap, permutation-importance, missing-data, and subgroup analyses were conducted in Python 3.12.14 using the same library versions, with SciPy 1.17.0 added.

## 3. Results

The results are presented in three stages corresponding to the evaluation framework. First, the two diagnostic formulations and their class distributions are described. Development-stage model stability and held-out discrimination are then reported, with held-out data kept separate from model selection. Finally, probability calibration, workload-aware operating characteristics, and internal temporal performance are examined to determine how the selected models affected case capture, missed cases, and referral workload.

### 3.1. Diagnostic Formulations and Class Balance

The source cohort comprised 311 records. Of these, 106 (34.1%) were classified as PTSD, 112 (36.0%) had no recorded post-event psychiatric diagnosis, and 93 (29.9%) had other non-PTSD clinical outcomes. These three outcome categories were mutually exclusive and collectively accounted for the entire source cohort (Table 3, Panel A).

The primary formulation compared 106 PTSD records with 112 records showing no recorded post-event psychiatric diagnosis, resulting in 218 records and a PTSD prevalence of 48.6%. The secondary formulation compared the same 106 PTSD records with 93 records showing other non-PTSD clinical outcomes, resulting in 199 records and a PTSD prevalence of 53.3% (Table 3, Panel B). The formulations represented distinct clinical contrasts but were not statistically independent because they shared the same PTSD records, whereas the comparator groups were mutually exclusive. Accordingly, performance differences between the formulations were interpreted descriptively and were not treated as formal evidence that one formulation was easier or more difficult to classify.

Class distributions were approximately balanced in both formulations. Supplementary experimental results are presented in Appendix A. Baseline child, family, and event characteristics across the three mutually exclusive source-cohort outcome groups are presented in Table A1.

Among the 106 PTSD records, 22 (20.8%) contained at least one additional recorded post-event diagnosis, most commonly a depressive diagnosis (18/106, 17.0%). Three records contained multiple additional diagnoses. No additional post-event diagnosis was recorded in 83 records (78.3%), while one record (0.9%) contained an unreadable-file note in the second diagnostic field and was reported separately. Within the 93 records with other non-PTSD clinical outcomes, the most frequent entries were depressive diagnoses (30/93, 32.3%), acute stress reaction (19/93, 20.4%), behavioral or conduct disorder entries (14/93, 15.1%), and anxiety-related entries (13/93, 14.0%). The detailed distribution of recorded co-diagnoses and other clinical entries is presented in Table A2.

### 3.2. Development Stability and Held-Out Discrimination

Repeated stratified cross-validation was used to characterize model stability within the development data and was not used as the final model-selection procedure. Stability estimates were calculated from the uncalibrated base-model probabilities and are reported as mean ± SD across 50 validation estimates. Model selection was performed separately using calibrated out-of-fold development predictions and the prespecified screening-oriented rule described in Section 2.4; the corresponding selection results are presented in Table A3. Held-out ROC-AUC and PR-AUC estimates were subsequently calculated from the continuous uncalibrated probabilities generated by the final base models fitted to the development data. In Table 4, boldface and an asterisk identify the model selected for each formulation before the held-out outcomes were examined.

In the primary formulation, random forest produced the highest mean cross-validation ROC-AUC and PR-AUC, with values of 0.6718 ± 0.0715 and 0.6965 ± 0.0772, respectively. However, histogram-based gradient boosting (HGB) was selected because it provided the highest calibrated out-of-fold specificity among the candidate models satisfying the prespecified sensitivity requirement of at least 0.85. When applied to the primary held-out set, the selected HGB pipeline yielded a ROC-AUC of 0.6190 (95% CI, 0.4472–0.7853) and a PR-AUC of 0.6578 (95% CI, 0.4786–0.8317).

In the secondary formulation, random forest again produced the highest mean cross-validation ROC-AUC and PR-AUC, with values of 0.6378 ± 0.1056 and 0.7035 ± 0.0815, respectively. LightGBM was nevertheless selected by the prespecified screening-oriented rule applied to the calibrated out-of-fold development predictions. On the secondary held-out set, the selected LightGBM pipeline yielded a ROC-AUC of 0.6441 (95% CI, 0.4475–0.8125) and a PR-AUC of 0.6805 (95% CI, 0.4837–0.8606).

The ROC-AUC confidence intervals of both selected models included 0.50. Exploratory tests against ROC-AUC = 0.50 yielded unadjusted *p*-values of 0.1734 for primary HGB and 0.1085 for secondary LightGBM; both Holm-adjusted *p*-values were 1.0000 (Table A4). These findings did not establish above-chance discrimination in the held-out samples. No formal pairwise superiority tests were prespecified, and the model-specific confidence intervals overlapped substantially. HGB and LightGBM were therefore retained as development-selected models for the subsequent calibration and workload analyses, rather than interpreted as universally superior classifiers. Results for all candidate models are presented in Table 4.

### 3.3. Calibration, Workload-Aware Thresholding, and Temporal Evaluation

Threshold-free discrimination alone did not establish whether predicted probabilities were reliable, how many referrals would be generated at clinically relevant operating points, or whether similar performance would be maintained under chronological separation. The development-selected models were therefore evaluated jointly in terms of probability calibration, workload-sensitive operating characteristics, and internal temporal robustness. These analyses were considered complementary dimensions of triage performance rather than independent evidence of clinical readiness.

#### 3.3.1. Probability Calibration

Sigmoid calibration reduced the Brier score of both development-selected models. In the primary formulation, the Brier score of histogram-based gradient boosting decreased from 0.3438 to 0.2537, corresponding to a ΔBrier of 0.0901. The calibration intercept moved from −1.1488 to −0.2074, while the calibration slope increased from 0.1431 to 0.4276. Although both estimates moved toward their ideal values of 0 and 1, respectively, the calibrated slope remained substantially below 1, suggesting that the predicted risks remained overly dispersed. Given the small held-out sample, this finding was interpreted cautiously.

The calibrated HGB Brier score of 0.2537 remained slightly higher than the prevalence-only reference value of 0.2496, resulting in a Brier skill score of −0.0165. Thus, sigmoid calibration improved the raw HGB probabilities but did not improve on the probability accuracy obtained by assigning the development-set PTSD prevalence to every held-out record.

In the secondary formulation, the LightGBM Brier score decreased from 0.3276 to 0.2401, corresponding to a ΔBrier of 0.0875. The calibration intercept moved from −0.1166 to −0.0383, and the calibration slope increased from 0.1729 to 1.1255. The calibrated Brier score was lower than the prevalence-only reference value of 0.2495, yielding a positive Brier skill score of 0.0376 and indicating a modest improvement over constant prevalence-based prediction. However, the secondary held-out set contained only 40 records, including 21 PTSD cases. The apparently favorable intercept and slope estimates were therefore considered exploratory rather than evidence of stable population-level calibration.

**Figure 2:**
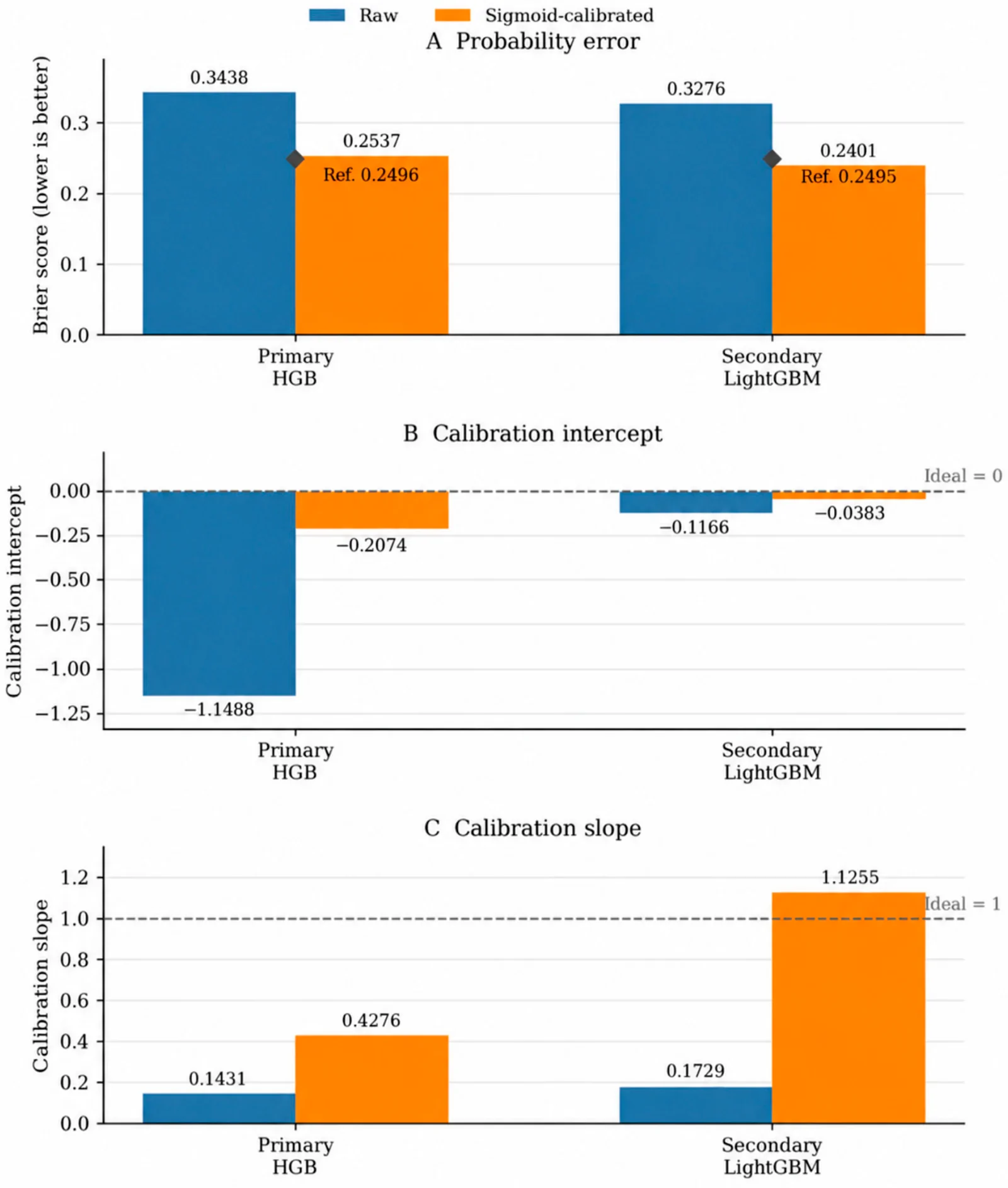
Held-out calibration before and after sigmoid calibration in the development-selected models: (**A**) Brier score; (**B**) calibration intercept; and (**C**) calibration slope. Diamonds indicate prevalence-only Brier references; dashed lines indicate ideal values.

Numerical calibration results are summarized in Table 5. Brier skill scores were calculated from unrounded quantities; recalculation using the displayed values may therefore produce small differences. Figure 2 visualizes the corresponding changes in probability error, calibration intercept, and calibration slope. Diamonds in Figure 2A indicate the prevalence-only Brier references, while the dashed lines in Figure 2B,C indicate the ideal intercept and slope values, respectively.

Taken together, sigmoid calibration reduced average probability error relative to the raw outputs in both formulations, but its value against the prevalence-only reference differed between the selected models. The primary model did not improve on this reference and retained a calibration slope substantially below 1, whereas the secondary model achieved only a modest positive Brier skill score. Given the small held-out samples, these intercept and slope estimates should be interpreted as exploratory and not as evidence of stable population-level calibration.

#### 3.3.2. Held-Out Operating Characteristics and Workload

At the deliberately permissive, fixed reference threshold of t = 0.05, both development-selected models flagged every held-out record. Sensitivity was therefore 1.0000 and specificity was 0.0000 in both formulations, with no reduction in referral workload. The fixed threshold achieved complete case capture only under an assess-all workload and did not provide a selective triage strategy.

The development-derived Rule A thresholds were applied unchanged to the held-out sets. In the primary formulation, the HGB threshold of 0.41 flagged 36 of 44 records (81.8%), captured 18 of 21 PTSD cases, and missed three. Sensitivity was 0.8571 (95% CI, 0.6366–0.9695), while specificity was 0.2174 (95% CI, 0.0746–0.4370), corresponding to an 18.2% reduction in referral workload relative to an assess-all strategy. In the secondary formulation, the LightGBM threshold of 0.44 flagged 30 of 40 records (75.0%), captured 18 of 21 PTSD cases, and missed three. Sensitivity was 0.8571 (95% CI, 0.6366–0.9695), specificity was 0.3684 (95% CI, 0.1629–0.6164), and referral workload was reduced by 25.0%.

The Rule B1 and Rule B2 criteria converged at the same threshold within each formulation. In the primary formulation, the threshold of 0.73 flagged only one record and missed 20 of 21 PTSD cases. The resulting positive predictive value of 1.0000 (95% CI, 0.0250–1.0000) was based on a single positive prediction. In the secondary formulation, the threshold of 0.70 flagged no records and missed all 21 PTSD cases; positive predictive value was therefore undefined. The specificity of 1.0000 at these restrictive operating points reflected near-empty or empty positive-prediction groups rather than reliable confirmation performance.

**Figure 3:**
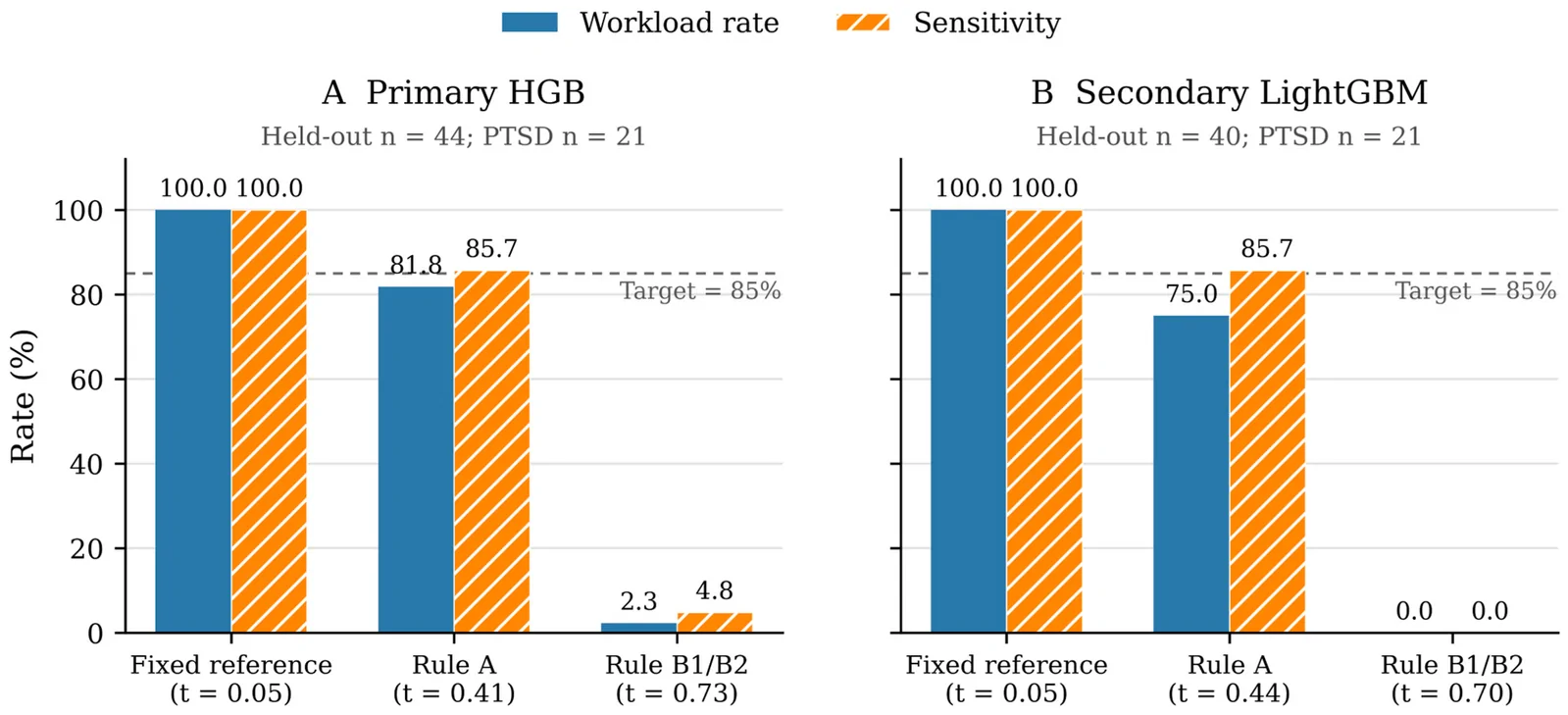
Held-out workload rate and sensitivity at the fixed reference and development-derived operating points for (**A**) the primary HGB model and (**B**) the secondary LightGBM model.

Threshold-specific counts and referral workload are presented in Table 6, Panel A, while the corresponding performance estimates and exact confidence intervals are reported in Panel B. NA denotes a metric that could not be calculated because the relevant prediction group was empty. Figure 3 compares workload rate and sensitivity across the fixed reference and development-derived operating points.

The exploratory comparison of development sensitivity requirements is presented in Table A5. In the primary formulation, increasing the requirement to 0.90 increased workload without capturing additional held-out PTSD cases; a requirement of 0.95 captured one additional case while flagging 42 of 44 records. In the secondary formulation, requirements of 0.90 and 0.95 captured 20 and 19 of 21 PTSD cases, respectively, with 33 of 40 records flagged in both cases. Higher development requirements therefore did not guarantee corresponding held-out sensitivity or a consistent improvement in case capture.

Taken together, none of the evaluated operating points simultaneously provided high PTSD case capture, high specificity, and low referral workload. Rule A retained the prespecified sensitivity target but required assessment of 81.8% and 75.0% of the primary and secondary held-out records, respectively. By contrast, the restrictive Rule B1/B2 thresholds reduced workload by 97.7% and 100.0% but missed 20 of 21 and 21 of 21 PTSD cases. Selection of an operating threshold should therefore be treated as a service-level governance decision informed by assessment capacity and tolerance for missed cases rather than as a purely statistical optimization problem.

#### 3.3.3. Internal Temporal Evaluation

Within the internal temporal evaluation, preprocessing, model selection, probability calibration, and threshold derivation were performed using only the earlier-period development records. The resulting pipelines were then applied unchanged to the later-period test records. This procedure selected HGB for the primary formulation and LightGBM for the secondary formulation without using later-period outcomes.

In the primary later-period set, HGB yielded a ROC-AUC of 0.6358 (95% CI, 0.4604–0.7917) and a PR-AUC of 0.5118 (95% CI, 0.3407–0.7467). Its development-derived Rule A threshold of 0.41 flagged 36 of 44 records (81.8%), captured 18 of 19 PTSD cases, and missed one. Sensitivity was 0.9474, specificity was 0.2800, positive predictive value was 0.5000, and negative predictive value was 0.8750.

In the secondary later-period set, LightGBM yielded a ROC-AUC of 0.6767 (95% CI, 0.4962–0.8672) and a PR-AUC of 0.6300 (95% CI, 0.4226–0.8492). Its development-derived Rule A threshold of 0.49 flagged 31 of 40 records (77.5%), captured 17 of 19 PTSD cases, and missed two. Sensitivity was 0.8947, specificity was 0.3333, positive predictive value was 0.5484, and negative predictive value was 0.7778. Sigmoid calibration reduced the Brier score from 0.3304 to 0.2438 for the primary HGB model and from 0.2755 to 0.2351 for the secondary LightGBM model.

Exploratory AUC tests yielded unadjusted *p*-values of 0.1091 for the selected primary HGB model and 0.0506 for the selected secondary LightGBM model. The corresponding Holm-adjusted *p*-values were 1.0000 and 0.9107. Among the other candidates, secondary HGB and XGBoost had unadjusted *p*-values of 0.0316 and 0.0186, respectively; their Holm-adjusted values were 0.6003 and 0.3713. None of the 20 AUC tests remained significant after Holm correction (Table A4).

Temporal discrimination and calibration results for all candidate models are presented in Table 7, Panel A, while performance at the development-derived Rule A thresholds is reported in Panel B. The analysis used strict chronological separation, and the models selected exclusively from the earlier-period development data are shown in bold.

#### 3.3.4. Bootstrap Stability Analysis

In the bootstrap analysis of the random-split development datasets, model and Rule A threshold selection varied across resamples. For the primary formulation, the originally selected HGB candidate was reselected in 31 of 100 replicates (31.0%). Its Rule A threshold, calculated in all replicates irrespective of which candidate was selected, had a median of 0.40 (IQR, 0.37–0.44; range, 0.21–0.49). For the secondary formulation, the originally selected LightGBM candidate was reselected in 18 of 100 replicates (18.0%). Its Rule A threshold, calculated in all replicates irrespective of which candidate was selected, had a median of 0.45 (IQR, 0.41–0.48; range, 0.29–0.56).

With the original held-out records retained, the repeatedly selected primary workflow had a median raw ROC-AUC of 0.5569 (IQR, 0.5155–0.6004), sensitivity of 0.8095 (IQR, 0.7619–0.9048), and workload proportion of 0.7955 (IQR, 0.7273–0.8864). Held-out sensitivity was at least 0.85 in 41 of 100 replicates. With the original held-out records retained, the repeatedly selected secondary workflow had a median raw ROC-AUC of 0.5927 (IQR, 0.5642–0.6247), sensitivity of 0.8571 (IQR, 0.8095–0.9524), and workload proportion of 0.8000 (IQR, 0.7250–0.9062). Held-out sensitivity was at least 0.85 in 60 of 100 replicates.

Model-specific threshold summaries and selection frequencies are presented in Table A6. Performance variability of the repeatedly selected workflow, with and without additional resampling of the held-out records, is presented in Table A7. These analyses describe sensitivity to the available development and evaluation samples and do not establish stable clinical operating characteristics.

#### 3.3.5. Exploratory Permutation Importance

In the primary HGB model, threat, paternal age, and family status produced the largest mean reductions in raw-score ROC-AUC after permutation: 0.0470, 0.0416, and 0.0390, respectively. These variables also produced the largest mean increases in calibrated Brier score, although their ordering differed.

In the secondary LightGBM model, maternal age, paternal age, and household composition produced the largest mean ROC-AUC reductions: 0.0432, 0.0298, and 0.0160, respectively. Family status, household composition, and threat produced the largest mean increases in calibrated Brier score. Rankings therefore depended on the model and scoring metric. Several estimates were near zero or negative, and permutation variability was substantial for several leading predictors. Complete results are presented in Table A8. These findings describe model-specific reliance on the recorded variables and do not establish clinically decisive or causal predictors of PTSD.

#### 3.3.6. Missing-Data Sensitivity Analysis

Missing-data handling affected discrimination and probability-error estimates in different ways (Table A9). Across the four strategies, primary raw ROC-AUC ranged from 0.5921 to 0.6273 and calibrated Brier score from 0.2480 to 0.2537. Primary Brier skill scores ranged from −0.0165 to 0.0066, indicating that the direction of the small difference from the prevalence-only reference depended on the handling strategy. Primary calibration slopes remained below 1, ranging from 0.4276 to 0.7486. Secondary raw ROC-AUC ranged from 0.6140 to 0.6667 and calibrated Brier score from 0.2394 to 0.2410, with Brier skill scores from 0.0339 to 0.0404. These descriptive differences do not establish the superiority of a particular strategy or eliminate uncertainty arising from missing data.

#### 3.3.7. Exploratory Subgroup Evaluation

Subgroup counts, sensitivity, and false-negative rates are presented in Table A10. In the primary random held-out evaluation, 3 of 15 PTSD records in the ≥12-year group were false negatives, compared with 0 of 4 in the <12-year group. The corresponding secondary counts were 2 of 15 and 1 of 4, respectively.

Male subgroups contained only two or three PTSD records across the evaluation sets. For example, the primary random held-out male false-negative rate was 1/3 (0.3333; 95% CI, 0.0084–0.9057), whereas the secondary random held-out male rate was 0/2 (0.0000; 95% CI, 0.0000–0.8419). Several abuse-type subgroups contained zero or one PTSD record, producing non-estimable measures or highly uncertain extreme estimates. These results do not establish reliable between-group differences, equivalent performance, or clinical safety across subgroups.

## 4. Discussion

This study assessed whether routinely recorded information could support PTSD-focused triage among children referred following sexual abuse. Four principal findings emerged. First, the available predictors provided only modest discrimination, with wide confidence intervals that included chance-level performance. Second, the sensitivity-oriented operating thresholds captured most PTSD cases but required assessment of most held-out records. Third, sigmoid calibration reduced probability error, although the primary model did not outperform the prevalence-only reference. Finally, internal temporal evaluation reproduced the same workload limitation. Taken together, these findings do not support autonomous diagnosis or clinical implementation of the models in their current form.

The results are broadly consistent with evidence that early post-trauma information may contribute to prediction of subsequent PTSD status or symptom trajectories [5,6,7,8]. The wider PTSD machine-learning literature has nevertheless shown considerable heterogeneity in study populations, predictor availability, outcome definitions, and validation procedures [9]. Studies specifically involving children exposed to sexual abuse have reported promising classification performance using artificial-intelligence-based decision-support and random forest approaches [12,13]. Direct numerical comparison with those studies would be inappropriate because of differences in cohort composition, outcome construction, prediction time, and evaluation design. The more modest held-out performance observed here suggests that the routinely documented variables available in these records may be insufficient for confident case-level separation in a forensic-referral setting.

ROC-AUC values of approximately 0.62–0.68 in the development-selected models reflected modest separation of the recorded outcome groups. None of the exploratory AUC tests remained significant after Holm correction (Table A4), and the findings did not establish reliable individual-level classification or clinical usefulness. Interpretation therefore considered discrimination jointly with calibration, missed PTSD cases, and referral workload.

The workload findings illustrate why discrimination metrics alone are inadequate for evaluating a triage model. At the development-derived Rule A thresholds, both selected models captured 18 of 21 PTSD cases but missed three cases. Achieving this sensitivity required 36 of 44 primary records and 30 of 40 secondary records to be flagged, corresponding to referral reductions in only 18.2% and 25.0%, respectively. Conversely, the restrictive Rule B1 and Rule B2 thresholds substantially reduced workload but missed nearly all PTSD cases. No evaluated operating point therefore achieved high case capture, high specificity, and a substantial reduction in assessment burden simultaneously. Threshold selection in this context is consequently a service-level governance decision that must reflect assessment capacity and the clinical consequences of missed cases rather than a purely statistical optimization problem.

Calibration provided a second distinction between statistical performance and potential clinical usefulness. Sigmoid calibration reduced the Brier score of both development-selected models, but improvement relative to the raw model does not necessarily indicate that the resulting probabilities are clinically informative. The primary model remained slightly worse than the prevalence-only reference, with a Brier skill score of −0.0165, whereas the secondary model achieved only a modest positive skill score of 0.0376. Calibration intercepts and slopes were also estimated from small held-out samples and should not be regarded as stable population parameters. These findings reinforce the importance of evaluating probability accuracy against an appropriate reference rather than interpreting calibrated model outputs as reliable risk estimates solely because calibration reduced the raw Brier score [10,11].

The two diagnostic formulations addressed different clinical contrasts. The primary formulation distinguished PTSD from the absence of a recorded post-event psychiatric diagnosis, whereas the secondary formulation distinguished PTSD from a heterogeneous group of other non-PTSD clinical outcomes. The slightly higher discrimination and Brier skill score in the secondary formulation should not be interpreted as evidence that this was an inherently easier or more clinically useful prediction task. The formulations shared the same PTSD records, used different comparator groups, and were not statistically independent. Moreover, the absence of a recorded diagnosis reflects the available documentation and does not establish the absence of psychiatric symptoms or subsequent disorder. The secondary formulation should likewise not be interpreted as a disorder-specific differential-diagnosis model.

Internal temporal evaluation provided a more demanding assessment than a random partition but did not materially change the clinical interpretation. Discrimination remained modest; the ROC-AUC confidence intervals included 0.50, and sensitivities of 0.9474 and 0.8947 required 81.8% and 77.5% of the later-period records to be flagged. Chronological separation therefore reproduced the trade-off between case capture and workload rather than resolving it. Because the earlier and later records originated from the same center, documentation system, and clinical setting, this analysis represents an internal temporal robustness assessment and does not establish transportability.

A methodological strength of the study was the separation of adaptive preprocessing, model selection, probability calibration, and threshold derivation from the held-out and later-period outcomes. Clinical performance was also considered jointly in terms of discrimination, calibration, missed cases, and referral workload. This evaluation framework reduces the risk of optimistic interpretation based on a single performance measure and is consistent with contemporary guidance for clinical prediction-model studies [10]. Nevertheless, strict internal evaluation cannot compensate for limitations in sample size, predictor availability, outcome documentation, or setting diversity.

Clinically, any future model derived from this data could be considered only as a prioritization layer supporting specialist assessment. Children referred following sexual abuse should not be excluded from assessment or care on the basis of an algorithmic score. Before clinical use could be considered, the approach would require external validation, prospective evaluation within the intended workflow, local recalibration, and explicit monitoring of referral burden, missed cases, and subgroup performance. The present findings establish the operational constraints that such evaluations would need to address rather than demonstrating readiness for implementation.

### Limitations

Several limitations should be considered when interpreting the findings. First, this retrospective single-center study relied on records generated during routine forensic psychiatric practice. Predictor documentation and diagnostic outcomes originated from the same specialist assessment and were not independently ascertained. Although post-event diagnosis fields and variables directly derived from diagnostic information were excluded from the model inputs, potential incorporation bias could arise if information included among the model predictors also contributed to the clinician’s diagnostic assessment. Such overlap could yield optimistic estimates of classification performance, and its extent could not be quantified from the available records. The psychiatrist-recorded DSM-5 diagnosis served as the retrospective reference standard; no additional structured research interview or independent diagnostic reassessment was performed. Outcome classification therefore remained dependent on the completeness of routine documentation and the clinical assessment process.

Twenty archived records were excluded because the reason for presentation could not be determined from the available documentation. Although the aggregate number and general reason for exclusion were identified, an individual-level exclusion log was not retained. More detailed exclusion criteria could therefore not be reported, and selection bias during cohort assembly cannot be completely ruled out. In addition, a stable person-level identifier was not consistently available in the analytical files, preventing reliable linkage of repeat records from the same child. If records from the same child were included in both development and evaluation samples, within-child dependence could have led to optimistic performance estimates. Chronological separation does not exclude this possibility because separate referrals for the same child could occur in different periods. The presence and extent of any resulting bias could not be determined from the available data. Future studies should retain a pseudonymized person-level identifier to support identification of repeat records and child-level separation during validation.

Second, the predictors were intended to represent routinely recorded information available during assessment, but exact variable-level documentation times could not be verified retrospectively for every record. Some variables may therefore have reflected information clarified later in the same assessment process. Prospective studies should define the intended assessment time point in advance and document predictor availability relative to that point.

Third, the predictor set was restricted to demographic, abuse-related, family, and contextual variables routinely documented during assessment. Standardized symptom scales, developmental assessments, previous trauma exposure, caregiver functioning, treatment history, and longitudinal clinical information were unavailable. This restriction may have limited discrimination and the ability to reduce false-positive referrals.

The permutation analysis provided global descriptions of the selected models rather than explanations of individual predictions. Modest discrimination, small evaluation samples, and dependence among predictors limit interpretation of the importance rankings. Permuting individual variables also does not preserve all relationships among predictors. Consequently, low or zero importance does not establish clinical irrelevance, and positive importance does not establish a causal effect or a validated clinical risk factor.

The missing-data sensitivity analysis was conditional on the original random partitions and selected model families. It did not address all possible imputation methods, propagate missing-value uncertainty through multiple imputations, or establish whether missingness depended on unobserved clinical information. Explicit unknown categories and missingness indicators may also reflect documentation practices. The comparisons therefore do not demonstrate that missing-data bias was eliminated.

Fourth, the two diagnostic formulations represented different clinical contrasts and excluded different comparator groups. Neither analytical cohort therefore represented the full clinical spectrum, and the observed class proportions should not be interpreted as population prevalences. The no-recorded-diagnosis group reflected the post-event diagnostic documentation and did not establish the absence of pre-existing mental disorders, psychiatric symptoms, or unmet clinical needs. The heterogeneous secondary comparator did not support disorder-specific differential-diagnosis claims.

Pre-existing disorders could affect discrimination and calibration through differences in case mix, without necessarily implying incorrect PTSD labels. If some true PTSD cases were undocumented, false-negative labels could reduce separation between groups, particularly if under-recording was unrelated to predictor values. However, the direction of AUC bias cannot be assumed when documentation errors depend on clinical presentation or predictor information. If misclassification primarily involved missed PTSD cases, a model could underestimate underlying PTSD probability despite apparently acceptable calibration against recorded labels. The net direction and magnitude of these effects could not be determined in this study without independent diagnostic reassessment.

Fifth, the numbers of records and PTSD events available for model development, calibration, threshold derivation, and evaluation were limited. The resulting confidence intervals were wide, and the additional bootstrap analysis quantified variability in model selection, Rule A thresholds, and evaluation performance rather than establishing sample adequacy. The analysis used 100 replicates per formulation and was conditional on the original random partition, limiting its ability to characterize rare instability patterns and its relevance to other populations. Record-level grouping prevented bootstrap copies of a source row from crossing validation folds but could not address unrecognized multiple records from the same child. Calibration intercepts, calibration slopes, and Brier skill scores estimated from the small held-out and temporal samples should likewise be interpreted as exploratory. The two formulations also shared the same PTSD records and were not statistically independent; differences between their performance estimates should therefore not be treated as formal comparative evidence. Model comparisons were also conditional on one fixed configuration per algorithm; alternative hyperparameter settings could change both performance estimates and the model selected by Rule A.

Synthetic data augmentation was not evaluated. Although such methods may affect model training, they would not increase the number of independent observed records available for evaluation or resolve the uncertainty associated with the small held-out samples.

Finally, the chronological evaluation was conducted within the same center, documentation system, and clinical setting as model development and cannot substitute for external validation. The additional subgroup analysis was descriptive and based on very few PTSD records in several groups, particularly males and less common abuse categories. Its pointwise confidence intervals do not establish equal performance, algorithmic fairness, or clinical safety across subgroups. The random and temporal evaluations also reused records and should not be pooled as independent evidence. The retrospective design did not assess how algorithmic prioritization would affect clinician decisions, referral delays, service workload, acceptability, patient outcomes, or safety in practice. Independent external validation, prospective workflow evaluation, local recalibration, and adequately supported subgroup assessment are required before clinical use can be considered.

## 5. Conclusions

In this retrospective classification study, routinely recorded information provided modest discrimination between clinician-recorded post-event diagnostic groups among children referred following sexual abuse. High PTSD case capture required flagging most records for assessment. The calibrated primary model had slightly higher probability error than a constant prediction based on development-set PTSD prevalence (Brier skill score, −0.0165) and a calibration slope of 0.4276. Similar findings under internal temporal separation indicated that the trade-off between sensitivity and referral workload was not specific to a random data partition. Additional bootstrap refitting showed variability in model selection, derived thresholds, and threshold-dependent performance. These results do not support autonomous diagnosis or clinical implementation in the current form. Any future application should be limited to prioritization in support of, rather than replacement for, specialist assessment. External validation, prospective workflow evaluation, local recalibration, and subgroup-level audit are required before clinical use can be considered.

## Figures and Tables

**Table 1:** Candidate predictor domains and formulation-specific missingness.

Predictor Domain	Candidate Predictors	Variable Types	Primary Missing/Unknown Range (%)	Secondary Missing/Unknown Range (%)
Child characteristics	Age at event; sex; biological/adopted status; school grade/status	Continuous and categorical	0.0–10.6	0.0–9.0
Maternal characteristics	Maternal education; employment; age	Continuous and categorical	6.4–19.7	5.0–14.1
Paternal characteristics	Paternal education; employment; age	Continuous and categorical	12.8–31.7	10.6–25.6
Family context	Parental consanguinity; family status; number of siblings; birth order; household composition; family psychiatric history	Discrete numeric and categorical	0.9–4.6	0.0–3.5
Event timing	Time from event to examination	Continuous	10.6	9.0
Abuse characteristics	Abuse type; threat; deception; number of abuse events	Categorical	6.4–22.0	6.0–23.1
Perpetrator relationship	Perpetrator relationship group	Nominal categorical	6.0	4.5

**Table 2:** Principal model configurations and class-weighting procedures.

Panel A. Fixed Model Configurations and Weighting Procedures.				
Model	Prespecified Fixed Configuration			Class-Imbalance Handling
L2 logistic regression	penalty = L2; C = 1.0; solver = liblinear; max_iter = 5000			class_weight = balanced; *w_c_* = *n*/(2*n_c_*)
Histogram-based gradient boosting	max_depth = 6; learning_rate = 0.08; max_iter = 500			No explicit class weighting
Random forest	n_estimators = 800; n_jobs = −1			class_weight = balanced_subsample
XGBoost	n_estimators = 800; learning_rate = 0.05; max_depth = 5; subsample = 0.90; colsample_bytree = 0.90; reg_lambda = 1.0			scale_pos_weight = *n*_negative_/*n*_positive_
LightGBM	n_estimators = 1200; learning_rate = 0.03; num_leaves = 63; subsample = 0.90; subsample_freq = 0; colsample_bytree = 0.90			scale_pos_weight = *n*_negative_/*n*_positive_
**Panel B. Formulation-Specific Class-Weight Values in the Final Development Fits.**				
**Model**	**Primary Random Development**	**Secondary Random Development**	**Primary Temporal Development**	**Secondary Temporal Development**
L2 logistic regression	w_0_ = 0.9775; w_1_ = 1.0235	w_0_ = 1.0743; w_1_ = 0.9353	w_0_ = 1.0000; w_1_ = 1.0000	w_0_ = 1.1042; w_1_ = 0.9138
Histogram-based gradient boosting	No explicit class weighting			
Random forest	Recomputed for each tree’s bootstrap sample			
XGBoost	1.0471	0.8706	1.0000	0.8276
LightGBM	1.0471	0.8706	1.0000	0.8276

**Table 3:** Source-cohort composition and diagnostic formulations.

Panel A. Mutually Exclusive Outcome Categories in the Source Cohort.					
Outcome Category			n	Source Cohort, %	
PTSD			106	34.1	
No recorded post-event psychiatric diagnosis			112	36.0	
Other non-PTSD clinical outcomes			93	29.9	
Total source cohort			311	100.0	
**Panel B. Binary Diagnostic Formulations Used for Model Development and Evaluation.**					
**Formulation**	**Total** **n**	**PTSD** **n**	**Comparator Group**	**Comparator** **n**	**PTSD Prevalence, %**
Primary	218	106	No recorded post-event psychiatric diagnosis	112	48.6
Secondary	199	106	Other non-PTSD clinical outcomes	93	53.3

**Table 4:** Development-stage cross-validation stability and held-out discrimination across candidate models.

Panel A. Primary Formulation.				
Model	CV ROC-AUC	CV PR-AUC	Held-Out ROC-AUC (95% CI)	Held-Out PR-AUC (95% CI)
Logistic regression	0.6485 ± 0.0800	0.6620 ± 0.0820	0.5445 (0.3483–0.7327)	0.6109 (0.4157–0.7945)
Histogram-based gradient boosting *	**0.6536 ± 0.0785**	**0.6674 ± 0.0858**	**0.6190 (0.4472–0.7853)**	**0.6578 (0.4786–0.8317)**
Random forest	0.6718 ± 0.0715	0.6965 ± 0.0772	0.5725 (0.3888–0.7537)	0.5630 (0.3823–0.7784)
XGBoost	0.6489 ± 0.0716	0.6641 ± 0.0723	0.5631 (0.3812–0.7367)	0.5934 (0.4080–0.7787)
LightGBM	0.6591 ± 0.0725	0.6710 ± 0.0823	0.5611 (0.3811–0.7299)	0.6132 (0.4326–0.7857)
**Panel B. Secondary Formulation.**				
Logistic regression	0.5839 ± 0.0995	0.6572 ± 0.0912	0.5990 (0.4025–0.7869)	0.5706 (0.3995–0.8073)
Histogram-based gradient boosting	0.6258 ± 0.0979	0.6792 ± 0.0851	0.6241 (0.4299–0.7969)	0.6502 (0.4603–0.8453)
Random forest	0.6378 ± 0.1056	0.7035 ± 0.0815	0.6429 (0.4369–0.8338)	0.5954 (0.4273–0.8480)
XGBoost	0.6045 ± 0.0946	0.6616 ± 0.0811	0.6190 (0.4166–0.7981)	0.5931 (0.4219–0.8285)
LightGBM *	**0.6135 ± 0.0993**	**0.6713 ± 0.0831**	**0.6441 (0.4475–0.8125)**	**0.6805 (0.4837–0.8606)**

CV denotes cross-validation; SD, standard deviation; CI, confidence interval; ROC-AUC, area under the receiver operating characteristic curve; and PR-AUC, average precision. Cross-validation results are reported as mean ± SD across 50 validation estimates. HGB denotes histogram-based gradient boosting, XGBoost denotes extreme gradient boosting, and LightGBM denotes light gradient boosting machine. Boldface and an asterisk (*) identify the model selected for each formulation using calibrated out-of-fold development predictions and the prespecified screening-oriented rule. These markings indicate development-stage selection and do not denote statistical significance or superiority on the held-out test set.

**Table 5:** Held-out calibration performance of the development-selected models.

Panel A. Probability-Error Measures.						
Formulation	Selected Model	Raw Brier Score	Calibrated Brier Score	ΔBrier	Prevalence-Only Brier Score	Brier Skill Score
Primary	Histogram-based gradient boosting	0.3438	0.2537	0.0901	0.2496	−0.0165
Secondary	LightGBM	0.3276	0.2401	0.0875	0.2495	0.0376
**Panel B. Calibration Intercept and Slope.**						
**Formulation**	**Selected Model**	**Raw Intercept**	**Calibrated Intercept**		**Raw Slope**	**Calibrated Slope**
Primary	Histogram-based gradient boosting	−1.1488	−0.2074		0.1431	0.4276
Secondary	LightGBM	−0.1166	−0.0383		0.1729	1.1255

**Table 6:** Held-out operating characteristics of the development-selected models.

Panel A. Threshold-Specific Counts and Referral Workload.								
Formulation	Operating Point	t	Workload n (%)	TP	FP	FN	TN	Referral Reduction %
Primary	Fixed reference	0.05	44 (100.0%)	21	23	0	0	0.0
Primary	Rule A	0.41	36 (81.8%)	18	18	3	5	18.2
Primary	Rule B1/B2	0.73	1 (2.3%)	1	0	20	23	97.7
Secondary	Fixed reference	0.05	40 (100.0%)	21	19	0	0	0.0
Secondary	Rule A	0.44	30 (75.0%)	18	12	3	7	25.0
Secondary	Rule B1/B2	0.70	0 (0.0%)	0	0	21	19	100.0
**Panel B. Threshold-Specific Performance with Exact 95% Confidence Intervals.**								
**Formulation**	**Operating Point**	**Sensitivity (95% CI)**	**Specificity (95% CI)**		**PPV (95% CI)**		**NPV (95% CI)**	
Primary	Fixed reference	1.0000 (0.8389–1.0000)	0.0000 (0.0000–0.1482)		0.4773 (0.3246–0.6331)		NA	
Primary	Rule A	0.8571 (0.6366–0.9695)	0.2174 (0.0746–0.4370)		0.5000 (0.3292–0.6708)		0.6250 (0.2449–0.9148)	
Primary	Rule B1/B2	0.0476 (0.0012–0.2382)	1.0000 (0.8518–1.0000)		1.0000 (0.0250–1.0000)		0.5349 (0.3765–0.6882)	
Secondary	Fixed reference	1.0000 (0.8389–1.0000)	0.0000 (0.0000–0.1765)		0.5250 (0.3613–0.6849)		NA	
Secondary	Rule A	0.8571 (0.6366–0.9695)	0.3684 (0.1629–0.6164)		0.6000 (0.4060–0.7734)		0.7000 (0.3475–0.9333)	
Secondary	Rule B1/B2	0.0000 (0.0000–0.1611)	1.0000 (0.8235–1.0000)		NA		0.4750 (0.3151–0.6387)	

**Table 7:** Internal temporal discrimination, calibration, and operating performance under strict chronological separation.

Panel A. Temporal Discrimination and Calibration.							
Formulation	Model	Date	Development/Test, n	ROC-AUC (95% CI)		PR-AUC (95% CI)	Brier Raw → Calibrated
Primary	Logistic regression	19 July 2018	174/44	0.6063 (0.4318–0.7655)		0.4858 (0.3227–0.7296)	0.2724 → 0.2447
Primary	HGB	19 July 2018	174/44	0.6358 (0.4604–0.7917)		0.5118 (0.3407–0.7467)	0.3304 → 0.2438
Primary	Random forest	19 July 2018	174/44	0.6411 (0.4669–0.7979)		0.5119 (0.3572–0.7627)	0.2428 → 0.2409
Primary	XGBoost	19 July 2018	174/44	0.6126 (0.4306–0.7769)		0.5121 (0.3360–0.7462)	0.3417 → 0.2413
Primary	LightGBM	19 July 2018	174/44	0.5874 (0.4132–0.7523)		0.4760 (0.3233–0.7064)	0.3290 → 0.2415
Secondary	Logistic regression	13 July 2018	159/40	0.5664 (0.3737–0.7387)		0.5109 (0.3419–0.7515)	0.2828 → 0.2539
Secondary	HGB	13 July 2018	159/40	0.6917 (0.4987–0.8747)		0.6444 (0.4397–0.8715)	0.2633 → 0.2383
Secondary	Random forest	13 July 2018	159/40	0.6053 (0.4261–0.7920)		0.5511 (0.3627–0.8094)	0.2524 → 0.2414
Secondary	XGBoost	13 July 2018	159/40	0.7043 (0.5320–0.8800)		0.6866 (0.4881–0.8920)	0.2568 → 0.2362
Secondary	LightGBM	13 July 2018	159/40	0.6767 (0.4962–0.8672)		0.6300 (0.4226–0.8492)	0.2755 → 0.2351
**Panel B. Operating Performance at the Development-Derived Rule A Thresholds.**							
**Formulation**	**Model**	**Rule A Threshold**	**Workload n (%)**	**Sensitivity**	**Specificity**	**PPV**	**NPV**
Primary	Logistic regression	0.42	34 (77.3%)	0.8421	0.2800	0.4706	0.7000
Primary	HGB	0.41	36 (81.8%)	0.9474	0.2800	0.5000	0.8750
Primary	Random forest	0.42	35 (79.5%)	0.9474	0.3200	0.5143	0.8889
Primary	XGBoost	0.41	35 (79.5%)	0.8947	0.2800	0.4857	0.7778
Primary	LightGBM	0.38	37 (84.1%)	0.9474	0.2400	0.4865	0.8571
Secondary	Logistic regression	0.44	37 (92.5%)	1.0000	0.1429	0.5135	1.0000
Secondary	HGB	0.47	32 (80.0%)	0.8421	0.2381	0.5000	0.6250
Secondary	Random forest	0.45	34 (85.0%)	1.0000	0.2857	0.5588	1.0000
Secondary	XGBoost	0.47	33 (82.5%)	0.8947	0.2381	0.5152	0.7143
Secondary	LightGBM	0.49	31 (77.5%)	0.8947	0.3333	0.5484	0.7778

Across both formulations, internal temporal evaluation reproduced the principal operational limitation observed in the random held-out analysis. The selected Rule A thresholds retained sensitivities of 0.9474 and 0.8947 but required 81.8% and 77.5% of the later-period records to be flagged, respectively. Both temporal ROC-AUC confidence intervals also included 0.50, indicating substantial uncertainty in the discrimination estimates. Chronological separation therefore did not eliminate the trade-off between PTSD case capture, specificity, and referral workload. Because the earlier and later records originated from the same center, documentation system, and clinical setting, these findings represent an internal temporal robustness assessment rather than external validation.

## Data Availability

The data are not publicly available because they contain sensitive clinical and forensic information concerning minors and are subject to ethical, privacy, and institutional restrictions. Requests for access may be directed to the corresponding author and will be considered only where permitted by the relevant ethics committee and institution.

## Abbreviations

The following abbreviations are used in this manuscript:

AUC	Area under the curve
BSS	Brier skill score
CI	Confidence interval
CV	Cross-validation
DSM-5	Diagnostic and Statistical Manual of Mental Disorders, Fifth Edition
FN	False negative
FP	False positive
HGB	Histogram-based gradient boosting
IQR	Interquartile range
LightGBM	Light Gradient Boosting Machine
ML	Machine learning
NA	Not available
NE	Not estimable
NPV	Negative predictive value
OOF	Out-of-fold
PPV	Positive predictive value
PR-AUC	Precision–recall area under the curve (reported as average precision)
PTSD	Post-traumatic stress disorder
ROC-AUC	Receiver operating characteristic area under the curve
SD	Standard deviation
TN	True negative
TP	True positive
XGBoost	Extreme Gradient Boosting

## Appendix A

Table A1 summarizes baseline characteristics across the three mutually exclusive source-cohort outcome groups defined in Section 2.1. Continuous variables are reported as median [interquartile range] with the number of non-missing records, whereas categorical variables are reported as n (%) of the corresponding group total. For both age at event and time from event to examination, group-specific missingness ranged from 6.6% to 14.3%. No values were imputed for this descriptive summary; imputation was performed only within model development, as described in the Methods. The “Other/not disclosed” abuse-type category combines the low-frequency not disclosed, exhibitionism, and abuse disclosed via social media categories.

**Table A1:** Baseline characteristics of the source cohort and diagnostic groups.

Characteristic	PTSD	No-Diagnosis	Other Clinical Outcome
N	106	112	93
Age at event, years, median [IQR]	13.53 [11.60–14.88] (n = 99)	13.64 [11.23–15.04] (n = 96)	14.02 [12.12–15.33] (n = 82)
Sex			
Female	93 (87.7%)	96 (85.7%)	77 (82.8%)
Male	13 (12.3%)	16 (14.3%)	16 (17.2%)
Time from event to examination, years, median [IQR]	1.00 [0.38–2.00] (n = 99)	0.58 [0.11–1.50] (n = 96)	0.50 [0.09–1.42] (n = 82)
School status			
Preschool	1 (0.9%)	1 (0.9%)	0 (0.0%)
Primary (grades 1–4)	10 (9.4%)	13 (11.6%)	10 (10.8%)
Middle (grades 5–8)	42 (39.6%)	36 (32.1%)	27 (29.0%)
High school (grades 9–12)	31 (29.2%)	32 (28.6%)	30 (32.3%)
Not enrolled/dropped out	21 (19.8%)	24 (21.4%)	24 (25.8%)
Special education	0 (0.0%)	1 (0.9%)	1 (1.1%)
Unknown	1 (0.9%)	5 (4.5%)	1 (1.1%)
Abuse type			
Contact, non-penetrative	47 (44.3%)	55 (49.1%)	43 (46.2%)
Penetration	50 (47.2%)	40 (35.7%)	35 (37.6%)
Verbal/non-contact	1 (0.9%)	5 (4.5%)	5 (5.4%)
Frotteurism/other contact	3 (2.8%)	1 (0.9%)	0 (0.0%)
Other/not disclosed	0 (0.0%)	2 (1.8%)	3 (3.2%)
Unknown	5 (4.7%)	9 (8.0%)	7 (7.5%)
Threat used			
Yes	42 (39.6%)	18 (16.1%)	22 (23.7%)
No	47 (44.3%)	70 (62.5%)	50 (53.8%)
Unknown	17 (16.0%)	24 (21.4%)	21 (22.6%)
Deception used			
Yes	19 (17.9%)	13 (11.6%)	17 (18.3%)
No	65 (61.3%)	73 (65.2%)	52 (55.9%)
Unknown	22 (20.8%)	26 (23.2%)	24 (25.8%)
Repeated event			
Single occurrence	51 (48.1%)	58 (51.8%)	49 (52.7%)
Multiple occurrences	48 (45.3%)	43 (38.4%)	35 (37.6%)
Unknown	7 (6.6%)	11 (9.8%)	9 (9.7%)
Perpetrator relationship			
Parent/step-parent	13 (12.3%)	11 (9.8%)	2 (2.2%)
Partner/spouse (of caregiver)	29 (27.4%)	29 (25.9%)	27 (29.0%)
Relative	13 (12.3%)	21 (18.8%)	8 (8.6%)
Acquaintance/other known	38 (35.8%)	32 (28.6%)	34 (36.6%)
Stranger	10 (9.4%)	9 (8.0%)	16 (17.2%)
Unknown	3 (2.8%)	10 (8.9%)	6 (6.5%)

**Table A2:** Recorded post-event co-diagnoses in PTSD records and clinical entries in the non-PTSD comparator group.

Panel A. PTSD Group (n = 106).		
Recorded Finding	n	%
Additional diagnosis status		
At least one additional recorded post-event diagnosis	22	20.8
No additional post-event diagnosis recorded	83	78.3
Unreadable file note in the second diagnostic field	1	0.9
Specific additional recorded diagnoses or clinical entries		
Depressive diagnoses	18	17.0
Social anxiety	1	0.9
Generalized anxiety	1	0.9
Separation anxiety	1	0.9
Adjustment disorder with depressive features	1	0.9
Behavioral disorder	1	0.9
Attention-deficit/hyperactivity disorder	1	0.9
Somatization	1	0.9
**Panel B. Other Non-PTSD Clinical Outcomes (n = 93).**		
**Recorded Diagnosis or Clinical Entry**	**n**	**%**
Depressive diagnoses	30	32.3
Acute stress reaction	19	20.4
Behavioral or conduct disorder entries	14	15.1
Anxiety disorder or anxiety unspecified	12	12.9
Attention-deficit/hyperactivity disorder	7	7.5
Adjustment disorder	5	5.4
ASB abbreviation as recorded	4	4.3
Medication-only entries without a specified diagnosis	3	3.2
Generalized anxiety	1	1.1
Acute stress disorder explicitly recorded	1	1.1
Past acute stress entry	1	1.1
Conversion disorder with an unspecified organic disease entry	1	1.1
Alcohol or substance dependence	1	1.1
Mood disorder unspecified	1	1.1
Anorexia nervosa	1	1.1
Activity disorder wording as recorded	1	1.1
Adverse psychological impact without a specified diagnosis	1	1.1

**Table A3:** Development-stage out-of-fold model comparison underlying model selection.

Formulation	Model	OOF ROC-AUC	OOF PR-AUC	OOF Brier	Rule A: Dev-OOF Sens.	Rule A: Dev-OOF Spec.	Rule A: Dev-OOF PPV
Primary (PTSD vs. no-diagnosis)	L2 logistic regression	0.6455	0.6090	0.2407	0.8588	0.2247	0.5141
**Primary (PTSD vs. no diagnosis)**	**Histogram-based gradient boosting ***	**0.6334**	**0.6201**	**0.2367**	**0.8824**	**0.2809**	**0.5396**
Primary (PTSD vs. no diagnosis)	Random forest	0.6404	0.6236	0.2375	0.8706	0.2584	0.5286
Primary (PTSD vs. no diagnosis)	XGBoost	0.6274	0.6007	0.2377	0.8941	0.2472	0.5315
Primary (PTSD vs. no diagnosis)	LightGBM	0.6324	0.6084	0.2370	0.8706	0.2360	0.5211
Secondary (PTSD vs. other outcome)	L2 logistic regression	0.5280	0.5507	0.2570	0.8824	0.1081	0.5319
Secondary (PTSD vs. other outcome)	Histogram-based gradient boosting	0.5908	0.6235	0.2425	0.8588	0.2162	0.5573
Secondary (PTSD vs. other outcome)	Random forest	0.5666	0.5955	0.2522	0.8941	0.1351	0.5429
Secondary (PTSD vs. other outcome)	XGBoost	0.5601	0.5752	0.2491	0.8706	0.2027	0.5564
**Secondary (PTSD vs. other outcome)**	**LightGBM ***	**0.6016**	**0.6375**	**0.2418**	**0.8588**	**0.2297**	**0.5615**

Development-stage model-selection results based on calibrated out-of-fold predictions are summarized in Table A3. Histogram-based gradient boosting and LightGBM, shown in bold and marked with an asterisk, were selected for the primary and secondary formulations, respectively, under the prespecified screening-oriented rule. Held-out outcomes were not used in this comparison.

Percentages in Table A2 were calculated separately within each outcome group, using 106 PTSD records and 93 records with other non-PTSD clinical outcomes as the denominators. Additional diagnostic categories were not mutually exclusive because three PTSD records contained multiple additional diagnoses. Repeated mentions of the same diagnosis within a record were counted once. Rows in Panel B were also not mutually exclusive because more than one diagnostic or clinical entry could be recorded for an individual record.

The absence of an additional recorded diagnosis reflected the available documentation and did not establish the absence of psychiatric comorbidity. One PTSD record containing an unreadable-file note in the second diagnostic field was reported separately. Medication-only and nonspecific clinical entries in the comparator group were retained as documented without assigning psychiatric diagnoses. The abbreviation ASB and the ambiguous activity-disorder wording were likewise preserved without further diagnostic interpretation.

**Table A4:** Exploratory tests of ROC-AUC against 0.50 in the random held-out and internal temporal evaluations.

Panel A. Random Held-Out Evaluation.				
Formulation	Model	ROC-AUC	Unadjusted *p*	Holm-Adjusted *p*
Primary	Logistic regression	0.5445	0.6287	1.0000
Primary	HGB	0.6190	0.1734	1.0000
Primary	Random forest	0.5725	0.4268	1.0000
Primary	XGBoost	0.5631	0.4786	1.0000
Primary	LightGBM	0.5611	0.4979	1.0000
Secondary	Logistic regression	0.5990	0.3042	1.0000
Secondary	HGB	0.6241	0.1736	1.0000
Secondary	Random forest	0.6429	0.1275	1.0000
Secondary	XGBoost	0.6190	0.2028	1.0000
Secondary	LightGBM	0.6441	0.1085	1.0000
**Panel B. Internal Temporal Evaluation.**				
Primary	Logistic regression	0.6063	0.2220	1.0000
Primary	HGB	0.6358	0.1091	1.0000
Primary	Random forest	0.6411	0.0973	1.0000
Primary	XGBoost	0.6126	0.1897	1.0000
Primary	LightGBM	0.5874	0.3249	1.0000
Secondary	Logistic regression	0.5664	0.4827	1.0000
Secondary	HGB	0.6917	0.0316	0.6003
Secondary	Random forest	0.6053	0.2499	1.0000
Secondary	XGBoost	0.7043	0.0186	0.3713
Secondary	LightGBM	0.6767	0.0506	0.9107

All tests were two-sided Wald tests using DeLong’s nonparametric variance estimator and the uncalibrated probabilities used for the ROC-AUC estimates in Table 4 and Table 7. Holm adjustment was applied jointly to all 20 tests. The primary formulation compared PTSD with no recorded post-event psychiatric diagnosis; the secondary formulation compared PTSD with other non-PTSD clinical outcomes. Evaluation sample sizes were 44 records for the primary formulation and 40 for the secondary formulation in each design. HGB denotes histogram-based gradient boosting. The tests were exploratory and did not influence development-stage selection.

**Table A5:** Exploratory comparison of development-stage sensitivity requirements and corresponding random held-out case capture and workload.

Formulation	Development Sensitivity Requirement	Selected Model	Probability Threshold	Held-Out PTSD Cases Captured	Held-Out Workload
Primary	≥0.85	HGB	0.41	18/21 (85.7%)	36/44 (81.8%)
Primary	≥0.90	Random forest	0.35	18/21 (85.7%)	40/44 (90.9%)
Primary	≥0.95	LightGBM	0.37	19/21 (90.5%)	42/44 (95.5%)
Secondary	≥0.85	LightGBM	0.44	18/21 (85.7%)	30/40 (75.0%)
Secondary	≥0.90	XGBoost	0.41	20/21 (95.2%)	33/40 (82.5%)
Secondary	≥0.95	HGB	0.40	19/21 (90.5%)	33/40 (82.5%)

Models and probability thresholds were selected separately for each sensitivity requirement using development data only. Consequently, comparisons reflect changes in the complete selection rule, including the selected model, rather than changes in the threshold of a single fixed model. Workload denotes the number of held-out records flagged for additional assessment. Each held-out set contained 21 records with PTSD. The additional comparisons were post hoc and descriptive.

**Table A6:** Stability of Rule A thresholds and model selection across full development-pipeline bootstrap replicates.

Formulation	Candidate	Selected, n (%)	Rule A Threshold, Median (IQR)	Threshold Min–Max
Primary	Logistic regression	13 (13.0)	0.40 (0.37–0.43)	0.27–0.47
Primary	HGB	31 (31.0)	0.40 (0.37–0.44)	0.21–0.49
Primary	Random forest	16 (16.0)	0.38 (0.36–0.42)	0.25–0.48
Primary	XGBoost	17 (17.0)	0.39 (0.35–0.42)	0.25–0.51
Primary	LightGBM	23 (23.0)	0.40 (0.36–0.43)	0.20–0.52
Secondary	Logistic regression	15 (15.0)	0.45 (0.41–0.48)	0.28–0.55
Secondary	HGB	18 (18.0)	0.46 (0.42–0.49)	0.28–0.57
Secondary	Random forest	29 (29.0)	0.44 (0.40–0.48)	0.32–0.54
Secondary	XGBoost	20 (20.0)	0.45 (0.41–0.48)	0.32–0.57
Secondary	LightGBM	18 (18.0)	0.45 (0.41–0.48)	0.29–0.56

Each formulation underwent 100 bootstrap repetitions of the development, calibration, model-selection, and Rule A threshold procedure. Selection frequencies sum to 100 within each formulation. Threshold summaries use all 100 thresholds derived for each candidate, including repetitions in which that candidate was not selected. IQR denotes interquartile range; HGB denotes histogram-based gradient boosting. The primary formulation compared PTSD with no recorded post-event psychiatric diagnosis, and the secondary formulation compared PTSD with other non-PTSD clinical outcomes. Different candidates’ thresholds were not pooled. The two formulations share the same 106 original PTSD records and are not independent samples.

**Table A7:** Variability in the performance of the repeatedly selected workflow across full development-pipeline bootstrap replicates.

Panel A. Evaluation on the Original Held-Out Records.		
Metric	Primary, Median (IQR)	Secondary, Median (IQR)
ROC-AUC (raw scores)	0.5569 (0.5155–0.6004)	0.5927 (0.5642–0.6247)
Average precision (raw scores)	0.5724 (0.5292–0.6098)	0.5980 (0.5705–0.6213)
Brier score (calibrated)	0.2523 (0.2471–0.2601)	0.2467 (0.2410–0.2530)
Brier skill score	−0.0028 (−0.0376–0.0171)	0.0147 (−0.0088–0.0382)
Sensitivity	0.8095 (0.7619–0.9048)	0.8571 (0.8095–0.9524)
Specificity	0.2174 (0.1304–0.3043)	0.2632 (0.1053–0.3684)
Workload proportion	0.7955 (0.7273–0.8864)	0.8000 (0.7250–0.9062)
**Panel B. Evaluation on Independently Resampled Held-out Records.**		
ROC-AUC (raw scores)	0.5534 (0.4853–0.6310)	0.5766 (0.5096–0.6460)
Average precision (raw scores)	0.5928 (0.5201–0.6767)	0.6278 (0.5547–0.7035)
Brier score (calibrated)	0.2530 (0.2423–0.2676)	0.2480 (0.2385–0.2592)
Brier skill score	−0.0085 (−0.0611–0.0280)	0.0034 (−0.0380–0.0449)
Sensitivity	0.8165 (0.7299–0.9058)	0.8775 (0.7765–0.9732)
Specificity	0.1818 (0.0825–0.3202)	0.2609 (0.0616–0.4000)
Workload proportion	0.7955 (0.7045–0.8920)	0.8000 (0.7000–0.9500)

Values are medians with interquartile ranges across 100 full development-pipeline bootstrap replicates. The selected model could differ between replicates. Panel A retains the original held-out records, whereas Panel B independently resamples those records in addition to refitting the development procedure. The original random development/test partition is retained throughout. ROC-AUC denotes area under the receiver operating characteristic curve; IQR denotes interquartile range. ROC-AUC and average precision use raw scores, whereas Brier scores and Rule A threshold metrics use calibrated probabilities. The prevalence-only reference uses the corresponding bootstrap development prevalence. Ranges are descriptive stability summaries, not replacement confidence intervals for the original model, and do not establish external validity or sample adequacy.

**Table A8:** Post hoc permutation importance of the 22 original predictors in the random held-out datasets.

Predictor	Primary: ROC-AUC Decrease	Secondary: ROC-AUC Decrease	Primary: Brier Increase	Secondary: Brier Increase
Age at event	0.0182 ± 0.0303	0.0050 ± 0.0388	0.0030 ± 0.0054	−0.0004 ± 0.0051
Sex	−0.0014 ± 0.0038	−0.0034 ± 0.0070	−0.0004 ± 0.0005	0.0000 ± 0.0007
Biological/adopted status	0.0000 ± 0.0000	0.0000 ± 0.0000	0.0000 ± 0.0000	0.0000 ± 0.0000
School grade/status	−0.0249 ± 0.0155	−0.0080 ± 0.0052	−0.0036 ± 0.0021	0.0006 ± 0.0006
Maternal education	0.0009 ± 0.0259	0.0000 ± 0.0047	0.0037 ± 0.0047	−0.0003 ± 0.0006
Paternal education	−0.0349 ± 0.0270	0.0136 ± 0.0171	−0.0035 ± 0.0055	0.0015 ± 0.0018
Maternal employment	0.0080 ± 0.0129	−0.0041 ± 0.0040	−0.0007 ± 0.0031	−0.0006 ± 0.0005
Paternal employment	0.0259 ± 0.0152	−0.0033 ± 0.0028	0.0013 ± 0.0008	0.0000 ± 0.0006
Maternal age	−0.0072 ± 0.0346	0.0432 ± 0.0260	0.0003 ± 0.0054	0.0025 ± 0.0031
Paternal age	0.0416 ± 0.0260	0.0298 ± 0.0451	0.0043 ± 0.0046	−0.0001 ± 0.0070
Parental consanguinity	−0.0051 ± 0.0019	−0.0089 ± 0.0050	−0.0007 ± 0.0004	−0.0001 ± 0.0001
Family status	0.0390 ± 0.0356	0.0117 ± 0.0402	0.0073 ± 0.0076	0.0032 ± 0.0053
Number of siblings	−0.0054 ± 0.0164	−0.0221 ± 0.0214	0.0010 ± 0.0021	0.0004 ± 0.0029
Birth order	−0.0016 ± 0.0215	0.0048 ± 0.0264	0.0015 ± 0.0034	0.0007 ± 0.0039
Household composition	0.0026 ± 0.0187	0.0160 ± 0.0110	0.0008 ± 0.0023	0.0029 ± 0.0013
Family psychiatric history	0.0000 ± 0.0000	0.0000 ± 0.0000	0.0000 ± 0.0000	0.0000 ± 0.0000
Time from event to examination	0.0183 ± 0.0354	0.0149 ± 0.0315	0.0011 ± 0.0056	0.0026 ± 0.0053
Abuse type	−0.0259 ± 0.0286	0.0121 ± 0.0119	−0.0019 ± 0.0050	0.0016 ± 0.0020
Threat	0.0470 ± 0.0472	0.0048 ± 0.0281	0.0093 ± 0.0102	0.0027 ± 0.0040
Deception	0.0129 ± 0.0149	−0.0098 ± 0.0136	−0.0003 ± 0.0017	−0.0012 ± 0.0022
Number of abuse events	−0.0429 ± 0.0245	0.0003 ± 0.0101	−0.0064 ± 0.0050	0.0009 ± 0.0012
Perpetrator relationship group	−0.0193 ± 0.0239	−0.0123 ± 0.0114	−0.0065 ± 0.0037	−0.0003 ± 0.0010

Values are means ± standard deviations across 100 permutations, rounded to four decimal places. The primary model was HGB and the secondary model was LightGBM. Positive values indicate deterioration in performance after permutation. Negative values indicate improved performance after permutation in the available sample and do not represent protective clinical effects. Standard deviations describe variability across permutations of the fixed held-out datasets, not sampling uncertainty. Importance values are specific to the fitted model, evaluation sample, and scoring metric.

**Table A9:** Sensitivity of discrimination and calibration to missing-data handling in the original random partitions.

Panel A. Discrimination and Probability Error.					
Formulation	Strategy	Raw ROC-AUC	Raw Average Precision	Calibrated Brier Score	Brier Skill Score
Primary	A	0.6190	0.6578	0.2537	−0.0165
Primary	B	0.6253	0.6676	0.2480	0.0065
Primary	C	0.5921	0.5717	0.2480	0.0066
Primary	D	0.6273	0.6960	0.2500	−0.0017
Secondary	A	0.6441	0.6805	0.2401	0.0376
Secondary	B	0.6667	0.7019	0.2410	0.0339
Secondary	C	0.6140	0.6301	0.2394	0.0404
Secondary	D	0.6466	0.6746	0.2408	0.0346
**Panel B. Calibration Intercept and Slope.**					
**Strategy**	**Primary Intercept**	**Primary Slope**	**Secondary Intercept**		**Secondary Slope**
A	−0.2074	0.4276	−0.0383		1.1255
B	−0.1577	0.7486	−0.0414		0.9107
C	−0.1858	0.6318	−0.0323		0.9406
D	−0.1826	0.5683	−0.0566		0.9986

The primary model was HGB and the secondary model was LightGBM. Strategies were A, the original numerical median imputation with categorical unknowns retained; B, numerical mean imputation with categorical handling unchanged; C, numerical median imputation with categorical unknowns replaced by the training-partition mode; and D, the original approach with numerical missingness indicators. All strategies used the same 174/44 primary and 159/40 secondary development/test partitions, with 21 PTSD records in each test set. Prevalence-only references used the corresponding development-set prevalence. Brier skill scores were calculated from unrounded quantities. Results are descriptive estimates rather than evidence of an optimal imputation strategy.

**Table A10:** Exploratory subgroup sensitivity and false-negative rates at the original development-derived Rule A thresholds.

Panel A. Primary Random Held-Out Evaluation: HGB, Threshold 0.41.					
Subgroup	N	PTSD n	TP/FN	Sensitivity (95% CI)	FNR (95% CI)
Age: <12 years	9	4	4/0	1.0000 (0.3976–1.0000)	0.0000 (0.0000–0.6024)
Age: ≥12 years	30	15	12/3	0.8000 (0.5191–0.9567)	0.2000 (0.0433–0.4809)
Age: Not recorded	5	2	2/0	1.0000 (0.1581–1.0000)	0.0000 (0.0000–0.8419)
Sex: Female	40	18	16/2	0.8889 (0.6529–0.9862)	0.1111 (0.0138–0.3471)
Sex: Male	4	3	2/1	0.6667 (0.0943–0.9916)	0.3333 (0.0084–0.9057)
Abuse: Contact, non-penetrative	25	13	11/2	0.8462 (0.5455–0.9808)	0.1538 (0.0192–0.4545)
Abuse: Penetration	14	7	7/0	1.0000 (0.5904–1.0000)	0.0000 (0.0000–0.4096)
Abuse: Verbal/non-contact	2	1	0/1	0.0000 (0.0000–0.9750)	1.0000 (0.0250–1.0000)
Abuse: Unknown	3	0	0/0	NE	NE
**Panel B. Secondary Random Held-out Evaluation: LightGBM, Threshold 0.44.**					
Age: <12 years	9	4	3/1	0.7500 (0.1941–0.9937)	0.2500 (0.0063–0.8059)
Age: ≥12 years	27	15	13/2	0.8667 (0.5954–0.9834)	0.1333 (0.0166–0.4046)
Age: Not recorded	4	2	2/0	1.0000 (0.1581–1.0000)	0.0000 (0.0000–0.8419)
Sex: Female	37	19	16/3	0.8421 (0.6042–0.9662)	0.1579 (0.0338–0.3958)
Sex: Male	3	2	2/0	1.0000 (0.1581–1.0000)	0.0000 (0.0000–0.8419)
Abuse: Contact, non-penetrative	21	10	8/2	0.8000 (0.4439–0.9748)	0.2000 (0.0252–0.5561)
Abuse: Penetration	13	9	8/1	0.8889 (0.5175–0.9972)	0.1111 (0.0028–0.4825)
Abuse: Verbal/non-contact	3	0	0/0	NE	NE
Abuse: Frotteurism/other contact	1	1	1/0	1.0000 (0.0250–1.0000)	0.0000 (0.0000–0.9750)
Abuse: Unknown	2	1	1/0	1.0000 (0.0250–1.0000)	0.0000 (0.0000–0.9750)
**Panel C. Primary Internal Temporal Evaluation: HGB, Threshold 0.41.**					
Age: <12 years	18	9	8/1	0.8889 (0.5175–0.9972)	0.1111 (0.0028–0.4825)
Age: ≥12 years	23	9	9/0	1.0000 (0.6637–1.0000)	0.0000 (0.0000–0.3363)
Age: Not recorded	3	1	1/0	1.0000 (0.0250–1.0000)	0.0000 (0.0000–0.9750)
Sex: Female	39	17	17/0	1.0000 (0.8049–1.0000)	0.0000 (0.0000–0.1951)
Sex: Male	5	2	1/1	0.5000 (0.0126–0.9874)	0.5000 (0.0126–0.9874)
Abuse: Contact, non-penetrative	28	12	12/0	1.0000 (0.7354–1.0000)	0.0000 (0.0000–0.2646)
Abuse: Penetration	8	2	2/0	1.0000 (0.1581–1.0000)	0.0000 (0.0000–0.8419)
Abuse: Verbal/non-contact	1	0	0/0	NE	NE
Abuse: Frotteurism/other contact	2	2	1/1	0.5000 (0.0126–0.9874)	0.5000 (0.0126–0.9874)
Abuse: Unknown	5	3	3/0	1.0000 (0.2924–1.0000)	0.0000 (0.0000–0.7076)
**Panel D. Secondary Internal Temporal Evaluation: LightGBM, Threshold 0.49.**					
Age: <12 years	11	9	7/2	0.7778 (0.3999–0.9719)	0.2222 (0.0281–0.6001)
Age: ≥12 years	25	9	9/0	1.0000 (0.6637–1.0000)	0.0000 (0.0000–0.3363)
Age: Not recorded	4	1	1/0	1.0000 (0.0250–1.0000)	0.0000 (0.0000–0.9750)
Sex: Female	34	17	15/2	0.8824 (0.6356–0.9854)	0.1176 (0.0146–0.3644)
Sex: Male	6	2	2/0	1.0000 (0.1581–1.0000)	0.0000 (0.0000–0.8419)
Abuse: Contact, non-penetrative	23	12	11/1	0.9167 (0.6152–0.9979)	0.0833 (0.0021–0.3848)
Abuse: Penetration	9	2	2/0	1.0000 (0.1581–1.0000)	0.0000 (0.0000–0.8419)
Abuse: Verbal/non-contact	1	0	0/0	NE	NE
Abuse: Frotteurism/other contact	2	2	2/0	1.0000 (0.1581–1.0000)	0.0000 (0.0000–0.8419)
Abuse: Other/not disclosed	1	0	0/0	NE	NE
Abuse: Unknown	4	3	2/1	0.6667 (0.0943–0.9916)	0.3333 (0.0084–0.9057)

Values are subgroup record counts and pointwise exact 95% Clopper–Pearson confidence intervals. TP/FN denotes the numbers of true-positive and false-negative PTSD records. Sensitivity and false-negative rate use the number of PTSD records within each subgroup as their denominator; NE denotes not estimable because no PTSD records were present. Age groups use the available, unimputed age at the event. Abuse-type labels follow the categories used in Table A1. The same records recur across subgroup domains, and the evaluation designs and diagnostic formulations are not independent; counts should not be pooled across domains, designs, or formulations. Zero observed false negatives do not establish zero underlying risk.
